# Supplementary material for: Do metacognitions contribute to pathological health anxiety? A systematic review and meta-analysis
Source: PLoS One. 2025 Jul 16;20(7):e0325563. doi: 10.1371/journal.pone.0325563 (PMC12266414; doi:10.1371/journal.pone.0325563)
Supplement: S2 Table — (DOCX) [file pone.0325563.s002.docx]

**S2 Table. Literature search.**
*Syntax of the search term*

| Database | Search term | Combination of search terms |
| --- | --- | --- |
| PubMed | ((Metacogn* OR “meta-cogn*”) AND (hypochondr* OR “illness anx*” OR “health anx*” OR cyberchondr* OR “disease phobia” OR “health worr*”)) OR ((Metacogn* OR “meta-cogn*”) AND (safety* OR reassurance* OR healthcare* OR “medical utilization” OR “illness behavior” OR “illness behaviour” OR scanning OR “body checking” OR “body-checking” OR care-* OR checking* OR avoid* OR “information avoid*”)) | 1. (Title/Abstract) AND 2. (Title/Abstract) OR 3. (Title/Abstract) AND |
| PsycINFo | (Metacogn* OR “meta-cogn*”) AND (hypochondr* OR “illness anx*” OR “health anx*” OR cyberchondr* OR “disease phobia” OR “health worr*”) OR (Metacogn* OR “meta-cogn*”) AND (safety* OR reassurance* OR healthcare* OR “medical utilization” OR “illness behavior” OR “illness behaviour” OR scanning OR “body checking” OR “body-checking” OR care-* OR checking* OR avoid* OR “information avoid*”) | 1. (Abstract) AND 2. (Abstract) OR 3. (Abstract) AND |
| Cochrane | Metacogn* OR meta NEXT cogn*  AND: hypochondr* OR illness NEXT anx* OR health NEXT anx* OR cyberchondr* OR “disease phobia” OR health NEXT worr*  OR: Metacogn* OR meta NEXT cogn*  AND: safety* OR reassurance* OR healthcare* OR “medical utilization” OR “illness behavior” OR “illness behaviour” OR scanning OR “body checking” OR “body checking” OR care-* OR checking* OR avoid* OR information NEXT avoid* | 1. (Title/Abstract/ Keyword) AND 2. (Title/Abstract/ Keyword) OR 3. (Title/Abstract/ Keyword) AND |
| MEDLINE | (Metacogn* OR “meta-cogn*”) AND (hypochondr* OR “illness anx*” OR “health anx*” OR cyberchondr* OR “disease phobia” OR “health worr*”) OR (Metacogn* OR “meta-cogn*”) AND (safety* OR reassurance* OR healthcare* OR “medical utilization” OR “illness behavior” OR “illness behaviour” OR scanning OR “body checking” OR “body-checking” OR care-* OR checking* OR avoid* OR “information avoid*”) | 1. (Title/Abstract) AND 2. (Title/Abstract) OR 3. (Title/Abstract) AND |
| Web of Science | TS=(Metacogn* OR “meta-cogn*”) AND  TS=(hypochondr* OR “illness anx*” OR “health anx*” OR cyberchondr* OR “disease phobia” OR “health worr*”) OR  TS=(Metacogn* OR “meta-cogn*”) AND (safety* OR reassurance* OR healthcare* OR “medical utilization” OR “illness behavior” OR “illness behaviour” OR scanning OR “body checking” OR “body-checking” OR care-* OR checking* OR avoid* OR “information avoid*”) | 1. (Topic) AND 2. (Topic) OR 3. (Topic) AND |
| PSYNDEX | ((Metakogn*) AND (Hypochondr* OR Krankheitsangst OR Krankheitssorgen OR Cyberchondr*)) OR ((Metakogn*) AND (Sicherheits* OR Arztbesuche OR Rückversicherung* OR Selbstuntersuchung OR hilfesuchend OR hilfemeidend OR Vermeidung*)) | 1. (Abstract) AND 2. (Abstract) OR 3. (Abstract) AND |
| ProQuest | ((Metacogn* OR “meta-cogn*”) AND (hypochondr* OR “illness anx*” OR “health anx*” OR cyberchondr* OR “disease phobia” OR “health worr*”)) OR ((Metacogn* OR “meta-cogn*”) AND (safety* OR reassurance* OR healthcare* OR “medical utilization” OR “illness behavior” OR “illness behaviour” OR scanning OR “body checking” OR “body-checking” OR care* OR checking* OR avoid* OR “information avoid*”)) | 1. (NOFT) AND 2. (NOFT) OR 3. (NOFT) AND |
| DNB | ((Metacogn* OR “meta-cogn*”) AND (hypochondr* OR “illness anx*” OR “health anx*” OR cyberchondr* OR “disease phobia” OR “health worr*”)) OR ((Metacogn* OR “meta-cogn*”) AND (safety* OR reassurance* OR healthcare* OR “medical utilization” OR “illness behavior” OR “illness behaviour” OR scanning OR “body checking” OR “body-checking” OR care-* OR checking* OR avoid* OR “information avoid*”)) | 1. (Alle Begriffe) UND 2. (Alle Begriffe) ODER 3. (Alle Begriffe) UND |

*Note.* TS = Topic.
