# Supplementary material for: Do metacognitions contribute to pathological health anxiety? A systematic review and meta-analysis
Source: PLoS One. 2025 Jul 16;20(7):e0325563. doi: 10.1371/journal.pone.0325563 (PMC12266414; doi:10.1371/journal.pone.0325563)
Supplement: S3 Table — (DOCX) [file pone.0325563.s003.docx]

**S3 Table. Excluded studies which might appear to meet the inclusion criteria.**

| Nr. | Study | Reason for exclusion |
| --- | --- | --- |
| 1 | Barahmand U. Meta-cognitive profiles in anxiety disorders. Psychiatry Res. 2009;169(3):240–243. | Data inaccessible by the first author; no response from the other authors |
| 2 | Buwalda FM, Bouman TK, Van Duijn MAJ. The effect of a psychoeducational course on hypochondriacal metacognition. Cogn Ther Res. 2008;32(5):689–701. | Data storage unit inaccessible by authors |
| 3 | Nasiri M, Mohammadkhani S, Akbari M, Alilou MM. The structural model of cyberchondria based on personality traits, health-related metacognition, cognitive bias, and emotion dysregulation. Front Psychiatry. 2023;13:960055. | No response from the authors |
| 4 | Taylor AR. An Investigation of the Relationship between Metacognition, Health Anxiety, and Obsessive Compulsive Disorder among an Outpatient Sample [dissertation]. Manchester (UK): University of Manchester; 2019. | No response from the author |
| 5 | Wells A. The metacognitive model of GAD: Assessment of meta-worry and relationship with DSM-IV generalized anxiety disorder. Cogn Ther Res. 2005;29(1):107–21. | Data storage unit inaccessible by authors |
| 6 | Wells A, Carter K. Further tests of a cognitive model of generalized anxiety disorder: Metacognitions and worry in GAD, panic disorder, social phobia, depression, and nonpatients. Behav Ther. 2001;32(1):85–102. | Data storage unit inaccessible by authors |
| 7 | Yang X, Gu D, Wu J, Liang C, Ma Y, Li J. Factors influencing health anxiety: the stimulus–organism–response model perspective. *Internet Res.* 2021;31(6):2033–2054. | No response from the authors |
