# Supplementary material for: Do metacognitions contribute to pathological health anxiety? A systematic review and meta-analysis
Source: PLoS One. 2025 Jul 16;20(7):e0325563. doi: 10.1371/journal.pone.0325563 (PMC12266414; doi:10.1371/journal.pone.0325563)
Supplement: S4 Table — (DOCX) [file pone.0325563.s004.docx]

**S4 Table. Data preparation.**
*Hierarchization and subscale pooling for the assessments of metacognitions.*

| Hierarchy^1^ | Measure | Relevant subscales^2^ | Computation | Construct | *k* (studies) |
| --- | --- | --- | --- | --- | --- |
| 1 | MCQ-HA  *Metacognitions about Health Questionnaire* | Beliefs about Biased thinking Beliefs that Thoughts can cause Illness, Beliefs that Thoughts are Uncontrollable | Subscale score Mean value of subscales | PMC NMC | 12 |
| 2 | MCHA  *Metacognition about Health Anxiety* | Positive consequences of thinking about illness Uncontrollability and interference, Negative consequences of thinking about illness | Subscale score Mean value of subscales | NMC PMC | 3 |
| 3a | MFK-30 mod.  *Metakognitionsfragebogen-30* | Uncontrollability / danger | Subscale score | NMC | 1 |
| 3b | WW-H mod.  *Why do people worry about health?* | Utility mod. | Subscale score | PMC | 1 |
| 4 | MCQ-30  *Meta-Cognitions Questionnaire-30* | Positive beliefs  Uncontrollability / danger | Subscale score Subscale score | PMC NMC | 8 |
| 5 | MCQ-65  *Meta-Cognitions Questionnaire-65* | Positive beliefs  Beliefs about the uncontrollability / danger | Subscale score Subscale score | PMC NMC | 3 |

*Note.* PMC = positive metacognitions, NMC = negative metacognitions, mod. = modified. MCQ-HA = Metacognitions Questionnaire-Health Anxiety, MCQ-30 = Metacognitions Questionnaire-30 (30 Items), MCQ-65 = Metacognitions Questionnaire-65 (65 Items), MKF-30 = Metacognitions Questionnaire-30 (short form in German) ^1^ If a study used 2 or more measuring instruments to record NMC and PMC, the most representative measuring instrument was selected. The list describes the order of representativeness of the questionnaires. ^2^ Labels correspond to the original paper of the development of the questionnaire, in some cases the subscale names are used in a slightly different way in the included studies.

*Hierarchization of the measurement instruments for health anxiety.*

| Hierarchy^1^ | Measure | Relevant subscales | Construct | *k* (studies) |
| --- | --- | --- | --- | --- |
| 1 | WI/WI-6  *Whiteley-Index* | Disease conviction Disease phobia Bodily preoccupation | HA | 13 |
| 2 | SHAI/SHAI-14  *Short Health Anxiety Inventory* | The feared likelihood of becoming ill The feared negative consequences of becoming ill | HA | 6 |
| 3 | HAQ  *Health Anxiety Questionnaire* | Health worry and preoccupation Fear of illness and death | HA | 2 |
| 4 | AnTI  *Anxious Thought Inventory* | Health worry | HA | 2 |

*Note.* Abbreviations. PMC = positive metacognitive beliefs, NMC = negative metacognitive beliefs, HA = health anxiety, SHAI-14 = Short Health Anxiety Inventory-14 (14 Items), SHAI = Short Healthy Anxiety Inventory (18 Items), WI = Whiteley Index (14 Items), WI-6 = Whiteley Index (6 Items)

^1^ If a study used 2 or more measuring instruments to record HA, the most representative measuring instrument was selected. The list describes the order of representativeness of the questionnaires.
