# Supplementary material for: Do metacognitions contribute to pathological health anxiety? A systematic review and meta-analysis
Source: PLoS One. 2025 Jul 16;20(7):e0325563. doi: 10.1371/journal.pone.0325563 (PMC12266414; doi:10.1371/journal.pone.0325563)
Supplement: S5 Table — (DOCX) [file pone.0325563.s005.docx]

**S5 Table. Sample characteristics.**

*Average health anxiety scores of the included studies.*

| Study | | *N* | Question-naire | Possible range of scores in the respective question-naires | Average health anxiety score *M* (*SD*) | Interpretation of averaged health anxiety score | Sample |  |
| --- | --- | --- | --- | --- | --- | --- | --- | --- |
| Melli et al. 2018 (1) | | 458 | HAQ | 21 to 84 | 64.69 (10.78) | - | clinical | |
| Melli et al. 2016 (2) | | 342 | HAQ | 21 to 84 | 32.47 (8.70) | - | analogue | |
| Airoldi et al. 2022 (3) | | 125 | SHAI-14 | 0 to 42 | 25.69 (6.08) | above cut-off, mild | analogue | |
| Nadeem et al. 2022 (4) | | 500 | SHAI-14 | 0 to 42 | 18.72 (8.77) | above cut-off, mild | analogue | |
| Akbari et al. 2022 (5) | | 541 | SHAI | 0 to 54 | 29.74 (6.13) | above cut-off | analogue | |
| Dai et al. 2018 (6) | | 1191 | SHAI | 0 to 54 | 11.73 (5.32) | below cut-off | analogue | |
| Penney et al. 2020 (7) | | 565 | SHAI | 0 to 54 | 16.47 (8.18) | above cut-off | analogue | |
| Rachor & Penney 2020 (8) | | 179 | SHAI | 0 to 54 | 20.27 (7.46) | above cut-off | analogue | |
| Bailey & Wells 2016 (9) | | 105 | WI (Likert) | 14 to 70 | 26.36 (9.36) | below cut-off | analogue | |
| Bailey & Wells 2013 (10) | | 351 | WI (Likert) | 14 to 70 | 26.37 (10.14) | below cut-off | analogue | |
| Bailey & Wells 2015 (11) | | 235 | WI (Likert) | 14 to 70 | not reported |  | analogue | |
| Barenbrügge et al. 2013 (12) | | 1264 | WI (dichotome) | 0 to 14 | 3.39 (2.87) | below cut-off | analogue | |
| Bouman & Meijer 1999 (13) | |  |  |  |  |  |  | |
|  | normal controls | 25 | WI (dichotome) | 0 to 14 | 2.0 (2.0) | below cut-off | analogue | |
|  | psychology students | 122 | WI (dichotome) | 0 to 14 | 2.7 (2.2) | below cut-off | analogue | |
|  | hypochondrial patients | 14 | WI (dichotome) | 0 to 14 | 10.9 (2.2) | above cut-off | clinical | |
| Kaur et al. 2011 (14) | | 158 | WI (Likert) | 14 to 70 | 13.94 (9.00) | below cut-off | analogue | |
| Solem et al. 2015 (15) | | 382 | WI (Likert) | 14 to 70 | 23.3 (7.70) | below cut-off | analogue | |
| Fergus & Bardeen 2019 (16) | | 785 | WI-6 | 6 to 30 | not reported | - | analogue | |
| Fergus & Spada 2017 (17) | | 337 | WI-6 | 6 to 30 | 13.14 (7.68) | - | analogue | |
| Fergus & Spada 2018 (18) | |  |  |  |  |  |  | |
|  | study 1 | 330 | WI-6 | 6 to 30 | 13.66 (5.20) | - | analogue | |
|  | study 2 | 331 | WI-6 | 6 to 30 | 13.7 (5.34) | - | analogue | |
| Fergus et al. 2022 (19) | |  |  |  |  |  |  | |
|  | non-latinx black | 123 | WI-6 | 6 to 30 | 14.53 (5.95) | - | analogue | |
|  | latinx | 104 | WI-6 | 6 to 30 | 14.53 (5.46) | - | analogue | |
|  | non-latinx white | 80 | WI-6 | 6 to 30 | 15.71 (6.72) | - | analogue | |
| Zheng et al. 2021 (20) | | 426 | WI-6 | 6 to 30 | not reported | - | analogue | |
| Cartwright-Hatton & Wells 1997 (21) | | 47 | AnTI | 6 to 24 | 9.5 (3.8) | - | analogue | |
| Wells & Papageorgiou 1998 (22) | | 105 | AnTI | 6 to 24 | 9.4 (not reported) | - | analogue | |

Abbreviations: HAQ = Health Anxiety Questionnaire, *note* response format of the Italian version used differs from the English version (shifting of the scale values), determined by personal correspondence with Gabriele Melli, Melli et al. (23) report average values of the Italian HAQ version of: *M* = 31.6, *SD* = 7.9 for men and *M* = 36, *SD* = 10.6 for women, HAI-14 = Short Health Anxiety Inventory-14 (14 Items), *note* a cut-off of 18 was suggested for analogue samples (‘healthy context’) and values are interpreted as follows: 0-27: no to mild health anxiety, 28-32: moderate health anxiety, 33-42: substantial health anxiety (24), SHAI = Short Healthy Anxiety Inventory (18 Items), *note* a cut-off of > 14 is interpreted as noticeable health anxious (25), WI = Whiteley Index (14 Items), *note* the cut-off depends on the response format (dichotome vs. 5-point Likert-scale), for WI (dichotome) a cut-off of 5 was used to discriminate persons with severe health anxiety from those without severe health anxiety (26) and for WI (Likert) a cut off score of 40 or above indicates the presence of hypochondriasis (27), WI-6 = Whiteley Index (6 Items), *note* Fergus et al. (28) report *M* = 21.28, *SD* = 5.54 for severe health anxiety and *M* = 13.49, *SD* = 5.60 for no severe health anxiety, there is no cut-off criterion, AnTI = Anxious Thought Inventory: the subscale health worry was used to determine the level of health anxiety, *note* Vázque Morejón et al. (29) report *M* = 12.66, *SD* = 4.84 in a clinical sample

**References**

1. Melli G, Bailey R, Carraresi C, Poli A. Metacognitive beliefs as a predictor of health anxiety in a self‐reporting Italian clinical sample. Clin Psychol Psychother. 2018 Mar;25(2):263–271.
2. Melli G, Carraresi C, Poli A, Bailey R. The role of metacognitive beliefs in health anxiety. Personal Individ Differ. 2016 Jan 1;89:80–85.
3. Airoldi S, Kolubinski DC, Nikčević AV, Spada MM. The relative contribution of health cognitions and metacognitions about health anxiety to cyberchondria: A prospective study. J Clin Psychol. 2022 May;78(5):809–820.
4. Nadeem F, Malik N, Atta M, Ullah I, Martinotti G, Pettorruso M, et al. Relationship between Health-Anxiety and Cyberchondria: Role of Metacognitive Beliefs. J Clin Med. 2022 May 5;11(9):2590.
5. Akbari M, Spada MM, Nikčević AV, Zamani E. The relationship between fear of COVID‐19 and health anxiety among families with COVID‐19 infected: The mediating role of metacognitions, intolerance of uncertainty and emotion regulation. Clin Psychol Psychother. 2021 Nov;28(6):1354–1366.
6. Dai L, Bailey R, Deng Y. The reliability and validity of the Chinese version of the Metacognitions about Health Questionnaire in college students. Qual Life Res. 2018 Apr 1;27(4):1099–1108.
7. Penney AM, Rachor GS, Deleurme KA. Differentiating the roles of intolerance of uncertainty and negative beliefs about worry across emotional disorders. J Exp Psychopathol. 2020 Oct 1;11(4):204380872097007.
8. Rachor GS, Penney AM. Exploring metacognitions in health anxiety and chronic pain: a cross-sectional survey. BMC Psychol. 2020 Dec;8(1):81.
9. Bailey R, Wells A. The contribution of metacognitive beliefs and dysfunctional illness beliefs in predicting health anxiety: An evaluation of the metacognitive versus the cognitive models. Clin Psychol. 2016 Nov 1;20(3):129–137.
10. Bailey R, Wells A. Does Metacognition Make a Unique Contribution to Health Anxiety When Controlling for Neuroticism, Illness Cognition, and Somatosensory Amplification? J Cogn Psychother. 2013;27(4):327–337.
11. Bailey R, Wells A. Metacognitive beliefs moderate the relationship between catastrophic misinterpretation and health anxiety. J Anxiety Disord. 2015 Aug 1;34:8–14.
12. Barenbrügge J, Glöckner-Rist A, Rist F. Positive und negative Metakognitionen über Krankheitssorgen. Psychotherapeut. 2013 Nov;58(6):560–568.
13. Bouman TK, Meijer KJ. A preliminary study of worry and metacognitions in hypochondriasis. Clin Psychol Psychother. 1999;6(2):96–101.
14. Kaur A, Butow P, Thewes B. Do Metacognitions Predict Attentional Bias in Health Anxiety? Cogn Ther Res. 2011 Dec;35(6):575–580.
15. Solem S, Borgejordet S, Haseth S, Hansen B, Håland Å, Bailey R. Symptoms of health anxiety in obsessive–compulsive disorder: Relationship with treatment outcome and metacognition. J Obsessive-Compuls Relat Disord. 2015 Apr;5:76–81.
16. Fergus TA, Bardeen JR. The Metacognitions Questionnaire–30: An Examination of a Bifactor Model and Measurement Invariance Among Men and Women in a Community Sample. Assessment. 2019 Mar;26(2):223–234.
17. Fergus TA, Spada MM. Cyberchondria: Examining relations with problematic Internet use and metacognitive beliefs. Clin Psychol Psychother. 2017 Nov;24(6):1322–1330.
18. Fergus TA, Spada MM. Moving toward a metacognitive conceptualization of cyberchondria: Examining the contribution of metacognitive beliefs, beliefs about rituals, and stop signals. J Anxiety Disord. 2018 Dec;60:11–9.
19. Fergus TA, Wilder K, Koester P, Kelley LP, Griggs JO. Metacognitive beliefs about uncontrollability relate most strongly to health anxiety among US‐based non‐Latinx White primary care patients: Comparing strength of relations with US‐based non‐Latinx Black and Latinx primary care patients. Clin Psychol Psychother. 2022 Jul;29(4):1331–1341.
20. Zheng H, Kyung Kim H, Joanna Sin SC, Theng YL. A theoretical model of cyberchondria development: Antecedents and intermediate processes. Telemat Inform. 2021 Oct;63:101659.
21. Cartwright-Hatton S, Wells A. Beliefs about Worry and Intrusions: The Meta-Cognitions Questionnaire and its Correlates. J Anxiety Disord. 1997 May;11(3):279–296.
22. Wells A, Papageorgiou C. Relationships between worry, obsessive–compulsive symptoms and meta-cognitive beliefs. Behav Res Ther. 1998 Sep;36(9):899–913.
23. Melli G, Coradeschi D, Smurra R. La versione italiana dell’Health Anxiety Questionnaire: Attendibilità e struttura fattoriale [The Italian version of Health Anxiety Questionnaire: Reliability and factorial analysis]. Psicoter Cogn E Comportamentale. 2007;13(1):37–48.
24. Österman S, Axelsson E, Lindefors N, Hedman-Lagerlöf E, Hedman-Lagerlöf M, Kern D, et al. The 14-item short health anxiety inventory (SHAI-14) used as a screening tool: appropriate interpretation and diagnostic accuracy of the Swedish version. BMC Psychiatry. 2022 Nov 14;22(1):701.
25. Rode S, Salkovskis P, Dowd H, Hanna M. Health anxiety levels in chronic pain clinic attenders. J Psychosom Res. 2006 Feb;60(2):155–161.
26. Hedman E, Lekander M, Ljótsson B, Lindefors N, Rück C, Andersson G, et al. Optimal Cut-Off Points on the Health Anxiety Inventory, Illness Attitude Scales and Whiteley Index to Identify Severe Health Anxiety. Elhai JD, editor. PLOS ONE. 2015 Apr 7;10(4):e0123412.
27. Gerdes TT, Noyes R, Kathol RG, Phillips BM, Fisher MM, Morcuende MA, et al. Physician recognition of hypochondriacal patients. Gen Hosp Psychiatry. 1996 Mar;18(2):106–112.
28. Fergus TA, Kelley LP, Griggs JO. Examining the Whiteley Index-6 as a screener for DSM-5 presentations of severe health anxiety in primary care. J Psychosom Res. 2019 Dec;127:109839.
29. Vázquez Morejón AJ, Vázquez-Morejón R, Muñoz-Fernández N. Anxious Thoughts Inventory (AnTI). Psychometric characteristics of an adaptation to the Spanish clinical population. Ansiedad Estrés. 2020 Jul;26(2–3):91–97.
