# Supplementary material for: Do metacognitions contribute to pathological health anxiety? A systematic review and meta-analysis
Source: PLoS One. 2025 Jul 16;20(7):e0325563. doi: 10.1371/journal.pone.0325563 (PMC12266414; doi:10.1371/journal.pone.0325563)
Supplement: S6 Table — (DOCX) [file pone.0325563.s006.docx]

**S6 Table. Risk of bias and Publication Bias: Detailed ROB analysis.**

*Risk of bias assessment*

|  | | | | **Power analysis** | | | **Description of sample** | | | | **Selection of participants** | | |  | **Replicability** | | | **Statistical methods** | | | | **ROB** | |
| --- | --- | --- | --- | --- | --- | --- | --- | --- | --- | --- | --- | --- | --- | --- | --- | --- | --- | --- | --- | --- | --- | --- | --- |
| Nr. | author (year) | | **Preregistration** | Any poweranalysis | Poweranalysis for relevant constructs | Poweranalysis described for recalculation | Total N | Gender distribution | Age | Educational background | Any in-/exclusion criteria | Dropout rates specified | Exclusion rates specified | **Reliability of the measures** | Replicability of recruitment process | Replicability of data collection process | Replicability of statistical analysis | Information if missing data (not) existent | Missing data described | Handling missing data described | Deviations in sample size described | low | high |
| 1 | Airoldi et al. (2022) | | no | no | N/A | N/A | yes | yes | yes | yes | yes | no | yes | yes | yes | yes | yes | yes | N/A | N/A | N/A | 12 | 2 |
| 2 | Akbari et al. (2021) | | no | no | N/A | N/A | yes | yes | yes | yes | yes | yes | yes | yes | yes | no | yes | no | no | no | no | 8 | 9 |
| 3 | Bailey & Wells (2013) | | no | no | N/A | N/A | yes | yes | yes | no | yes | no | yes | no | yes | no | yes | no | no | no | no | 6 | 11 |
| 4 | Bailey & Wells (2015a) | | no | no | N/A | N/A | yes | yes | yes | no | yes | yes | yes | yes | yes | no | yes | no | no | no | no | 7 | 10 |
| 5 | Bailey & Wells (2016a) | | no | no | N/A | N/A | yes | yes | yes | no | yes | no | yes | no | no | yes | yes | no | no | no | no | 7 | 10 |
| 6 | Barenbrügge et al. (2013) | | no | no | N/A | N/A | yes | yes | yes | yes | yes | no | yes | no | yes | yes | yes | yes | N/A | N/A | N/A | 11 | 3 |
| 7 | Bouman & Meijer (1999) | | no | no | N/A | N/A | yes | yes | yes | yes | yes | yes | yes | no | no | no | yes | no | no | no | no | 6 | 11 |
| 8 | Cartwright-Hatton & Wells (1997) | | no | no | N/A | N/A | yes | yes | yes | no | yes | no | yes | no | no | no | yes | no | no | no | no | 4 | 13 |
| 9 | Dai et al. (2018) | | no | no | N/A | N/A | yes | yes | yes | no | yes | yes | yes | yes | yes | no | yes | yes | N/A | N/A | N/A | 8 | 6 |
| 10 | Fergus & Bardeen (2019) | | no | no | N/A | N/A | yes | yes | yes | no | yes | no | yes | yes | yes | yes | yes | no | no | no | no | 8 | 9 |
| 11 | Fergus & Spada (2017) | | no | yes | no | no | yes | yes | yes | yes | yes | yes | yes | yes | yes | yes | yes | no | no | no | no | 10 | 9 |
| 12 | Fergus & Spada (2018) | |  |  |  |  |  |  |  |  |  |  |  |  |  |  |  |  |  |  |  |  |  |
|  |  | study 1 | no | no | N/A | N/A | yes | yes | yes | no | yes | no | no | yes | yes | yes | yes | yes | yes | yes | no | 11 | 6 |
| 13 |  | study 2 | no | no | N/A | N/A | yes | yes | yes | yes | yes | no | no | yes | yes | yes | yes | yes | N/A | N/A | N/A | 10 | 4 |
| 14 | Fergus et al. (2022) | | no | yes | yes | yes | yes | yes | yes | no | yes | no | yes | yes | yes | yes | yes | yes | yes | yes | yes | 17 | 2 |
| 15 | Kaur et al. (2011) | | no | no | N/A | N/A | yes | yes | yes | no | yes | yes | yes | no | yes | yes | yes | no | no | no | no | 7 | 10 |
| 16 | Melli et al. (2018) | | no | no | N/A | N/A | yes | yes | yes | yes | yes | no | yes | yes | yes | yes | yes | no | no | no | no | 9 | 8 |
| 17 | Melli et al. (2016) | | no | no | N/A | N/A | yes | yes | yes | yes | yes | yes | yes | yes | yes | yes | yes | no | no | no | no | 9 | 8 |
| 18 | Nadeem et al. (2022) | | no | yes | yes | yes | yes | yes | no | yes | yes | no | yes | yes | no | yes | yes | yes | N/A | N/A | yes | 13 | 4 |
| 19 | Penney et al. (2020) | | no | yes | no | yes | yes | yes | yes | no | yes | yes | yes | yes | yes | yes | yes | yes | no | no | no | 10 | 7 |
| 20 | Rachor & Penney (2020) | | no | no | N/A | N/A | yes | yes | yes | no | yes | no | yes | yes | yes | yes | yes | no | no | no | no | 9 | 8 |
| 21 | Solem et al. (2015) | | no | no | N/A | N/A | yes | yes | yes | no | yes | yes | yes | yes | yes | yes | yes | yes | no | no | yes | 11 | 6 |
| 22 | Wells & Papageorgiou (1998) | | no | no | N/A | N/A | yes | yes | yes | no | yes | yes | yes | no | yes | yes | yes | no | no | no | no | 7 | 9 |
| 23 | Zheng et al. (2021) | | no | no | N/A | N/A | yes | yes | no | yes | yes | no | yes | yes | yes | yes | yes | no | no | no | no | 10 | 7 |
|  | Total low ROB (yes) | | 0 | 4 | 2 | 3 | 23 | 23 | 21 | 10 | 19 | 8 | 6 | 16 | 19 | 17 | 23 | 9 | 2 | 2 | 3 |  |  |
|  | Total high ROB (no) | | 23 | 19 | 2 | 1 | 0 | 0 | 2 | 13 | 3 | 15 | 15 | 7 | 4 | 6 | 0 | 14 | 16 | 16 | 16 |  |  |
| *Note.*  ROB = risk of bias, total low ROB (yes) = total categories with low risk of bias, Total high ROB (no) = total categories with high Risk of bias | | | | | | | | | | | | | | | | | | | | | | | |
