# Supplementary material for: Do metacognitions contribute to pathological health anxiety? A systematic review and meta-analysis
Source: PLoS One. 2025 Jul 16;20(7):e0325563. doi: 10.1371/journal.pone.0325563 (PMC12266414; doi:10.1371/journal.pone.0325563)
Supplement: S7 Table — (DOCX) [file pone.0325563.s007.docx]

**S7 Table. Moderator analyses.**

*Subgroup analyses for assessments of metacognitions and health anxiety as well as analysis strategy.*

|  | *k* | τ | τ² | cor | *Q* | *I²* | Differences for PMCs and NMCs |
| --- | --- | --- | --- | --- | --- | --- | --- |
| **MCQ-HA vs. MCQ-30, MCQ-65 and MKF-30** | | | | | | | |
| MCQ-HA | 11 | 0.18 | 0.03 | 0.44 (0.33 - 0.53) | 116.57 | 91.4% (86.7% - 94.5%) | PMCs (*p* = 0.07) |
| MCQ-30 etc. | 8 | 0.14 | 0.02 | 0.32 (0.2 - 0.42) | 55.11 | 87.3% (77.2% - 92.9%) |  |
| MCQ-HA | 12 | 0.15 | 0.02 | 0.5 (0.41 - 0.57) | 94.73 | 88.4% (81.6% - 92.7%) | NMCs (*p* = 0.348) |
| MCQ-30 etc. | 9 | 0.20 | 0.04 | 0.55 (0.43 - 0.66) | 178.29 | 95.5% (93.3% - 97%) |  |
| **SHAI/SHAI-14 vs. WI/WI-6** | | | | | | | |
| SHAI | 6 | 0.26 | 0.07 | 0.35 (0.09 - 0.57) | 146.88 | 96.6% (94.6% - 97.9%) | PMCs (*p* = 0.743) |
| WI | 12 | 0.15 | 0.02 | 0.38 (0.29 - 0.47) | 116.72 | 90.6% (85.5% - 93.9%) |  |
| SHAI | 6 | 0.13 | 0.02 | 0.49 (0.37 - 0.59) | 53.13 | 90.6% (82.3% - 95%) | NMCs (*p* = 0.159) |
| WI | 13 | 0.18 | 0.03 | 0.57 (0.48 - 0.64) | 184.18 | 93.5% (90.6% - 95.5%) |  |
| **singular correlations vs. mean correlations** | | | | | | | |
| singular | 19 | 0.18 | 0.03 | 0.38 (0.3 - 0.45) | 222.39 | 91.9% (88.8% - 94.2%) | PMCs (*p* = 0.236) |
| mean | 2 | 0.13 | 0.02 | 0.27 (-0.75 - 0.91) | 9.30 | 89.3% (59.8% - 97.1%) |  |
| singular | 9 | 0.14 | 0.02 | 0.53 (0.44 - 0.62) | 49.08 | 83.7% (70.6% - 91%) | NMCs (*p* = 0.99) |
| mean | 13 | 0.18 | 0.03 | 0.53 (0.45 - 0.61) | 291.69 | 95.9% (94.3% - 97%) |  |

*Note.* “Values (95% Confidence Intervals)”, Abbreviations: PMC = positive metacognitive beliefs, NMC = negative metacognitive beliefs, MCQ-HA = Metacognitions Questionnaire-Health Anxiety, MCQ-30 = Metacognitions Questionnaire-30 (30 Items), MCQ-65 = Metacognitions Questionnaire-65 (65 Items), MKF-30 = Metacognitions Questionnaire-30 (short form in German), SHAI-14 = Short Health Anxiety Inventory-14 (14 Items), SHAI = Short Healthy Anxiety Inventory (18 Items), WI = Whiteley Index (14 Items), WI-6 = Whiteley Index (6 Items)
