# Supplementary material for: Do metacognitions contribute to pathological health anxiety? A systematic review and meta-analysis
Source: PLoS One. 2025 Jul 16;20(7):e0325563. doi: 10.1371/journal.pone.0325563 (PMC12266414; doi:10.1371/journal.pone.0325563)
Supplement: S1 Text — (DOCX) [file pone.0325563.s008.docx]

**S1 Text. References of articles included in the meta-analysis.**

This bibliography contains only the references that were used in the meta-analysis but are not discussed in the paper.

1. Airoldi S, Kolubinski DC, Nikčević AV, Spada MM. The relative contribution of health cognitions and metacognitions about health anxiety to cyberchondria: A prospective study. J Clin Psychol. 2022;78(5):809–820.
2. Solem S, Borgejordet S, Haseth S, Hansen B, Håland Å, Bailey R. Symptoms of health anxiety in obsessive–compulsive disorder: Relationship with treatment outcome and metacognition. J Obsessive Compuls Relat Disord. 2015;5:76–81
3. Wells A, Papageorgiou C. Relationships between worry, obsessive–compulsive symptoms and meta-cognitive beliefs. Behav Res Ther. 1998;36(9):899–913.
