# Supplementary material for: Do metacognitions contribute to pathological health anxiety? A systematic review and meta-analysis
Source: PLoS One. 2025 Jul 16;20(7):e0325563. doi: 10.1371/journal.pone.0325563 (PMC12266414; doi:10.1371/journal.pone.0325563)
Supplement: S1 Appendix — (DOCX) [file pone.0325563.s009.docx]

**S1 Appendix. Sensitivity analyses.**

**Sensitivity analyses**

The outlier analyses detected *k* = 4 studies for PMC [1–4] and *k* = 6 studies for NMC [3–8] as outliers. The random effects model without the outliers revealed a marginal higher effect for PMC and health anxiety, *r* = .35 (*p* < .0001; 95%-CI: .29; 0.41), and decreased but still high heterogeneity, *I*^2^ = 78.9% (95%-CI: 67.3; 86.4). The Baujat plot demonstrated that *k* = 2 studies were mainly contributing to heterogeneity [3,4]. The leave-one-out analysis showed a marginal change of the effect to *r* = .34 (95%-CI: .28; .41; *I*^2^ = 88%) excluding the study from Nadeem et al. [4] and as well as a slight increase of the effect to *r* = .37 (95%-CI: .30; .44; *I*^2^ = 91%) without the study by Barenbrügge et al. [3] . For the correlation between NMC and health anxiety, the magnitude of the estimated effect without the outlying studies stayed the same as with the outliers, *r* = .52 (*p* < .0001; 95%-CI: .47; .56). But there was a decrease in heterogeneity, *I*^2^ = 72.2% (95%-CI: 54.8; 82.9). The Baujat plot also revealed *k* = 2 studies [3,7] relevant contributions to heterogeneity. For NMC the leave-one-out analysis only showed minor differences of the effects without Barenbrügge et al. [3, *r* = .51, 95%-CI: .45; .56; *I*^2^ = 87%] as well as without Dai et al. [7, *r* = .53, 95%-CI .46; .58; *I*^2^ = 93%].

**References**

1. Akbari M, Spada MM, Nikčević AV, Zamani E. The relationship between fear of COVID‐19 and health anxiety among families with COVID‐19 infected: The mediating role of metacognitions, intolerance of uncertainty and emotion regulation. Clin Psychol Psychother. 2021 Nov;28(6):1354–1366.

2. Bailey R, Wells A. Metacognitive beliefs moderate the relationship between catastrophic misinterpretation and health anxiety. J Anxiety Disord. 2015 Aug 1;34:8–14.

3. Barenbrügge J, Glöckner-Rist A, Rist F. Positive und negative Metakognitionen über Krankheitssorgen. Psychotherapeut. 2013 Nov;58(6):560–568.

4. Nadeem F, Malik N, Atta M, Ullah I, Martinotti G, Pettorruso M, et al. Relationship between Health-Anxiety and Cyberchondria: Role of Metacognitive Beliefs. J Clin Med. 2022 May 5;11(9):2590.

5. Bailey R, Wells A. Does Metacognition Make a Unique Contribution to Health Anxiety When Controlling for Neuroticism, Illness Cognition, and Somatosensory Amplification? J Cogn Psychother. 2013;27(4):327–337.

6. Bailey R, Wells A. The contribution of metacognitive beliefs and dysfunctional illness beliefs in predicting health anxiety: An evaluation of the metacognitive versus the cognitive models. Clin Psychol. 2016 Nov 1;20(3):129–137.

7. Dai L, Bailey R, Deng Y. The reliability and validity of the Chinese version of the Metacognitions about Health Questionnaire in college students. Qual Life Res. 2018 Apr 1;27(4):1099–1108.

8. Melli G, Carraresi C, Poli A, Bailey R. The role of metacognitive beliefs in health anxiety. Personal Individ Differ. 2016 Jan 1;89:80–85.

**Baujat Plots**

|  | Positive metacognitions (PMC) | Negative metacognitions (NMC) | |  |
| --- | --- | --- | --- | --- |
|  | **Supplementary Figure** | **Supplementary Figure** | |  |
| Health anxiety (HA) | 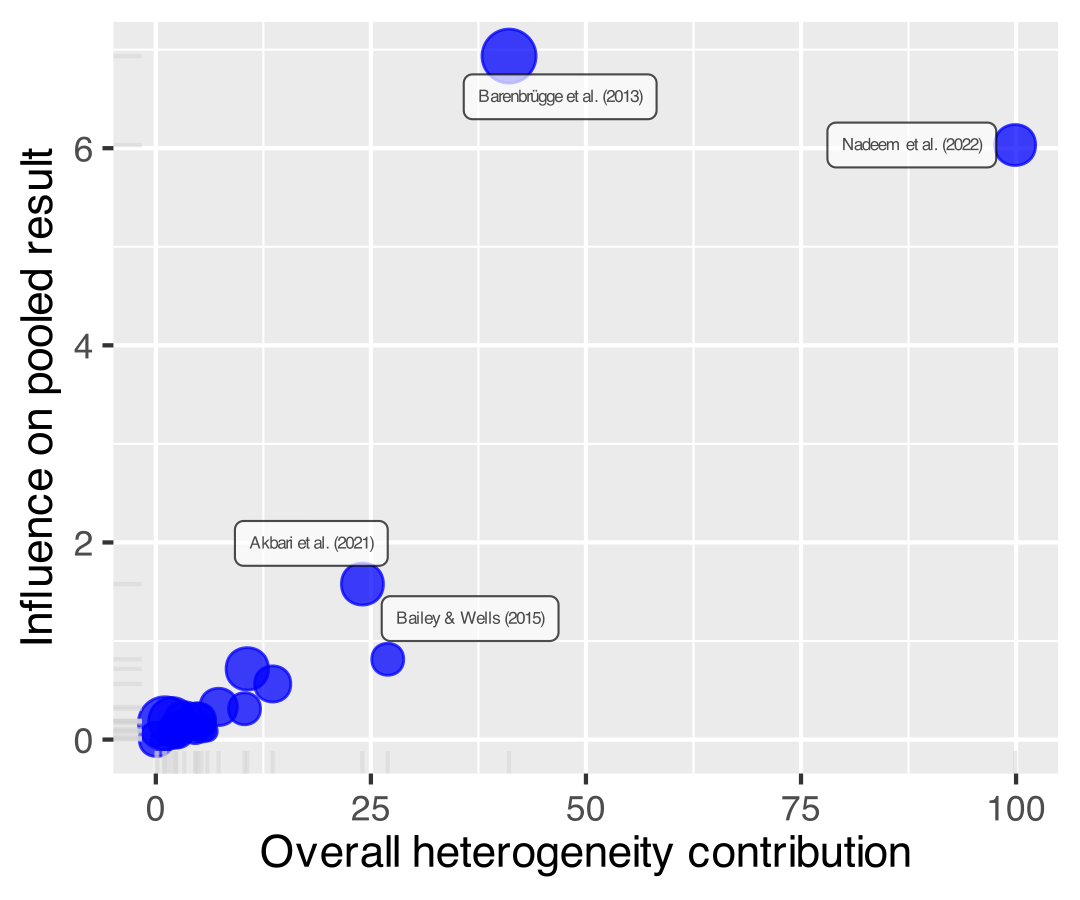  *Note.* Baujat Plot for positive metacognitions and health anxiety | | 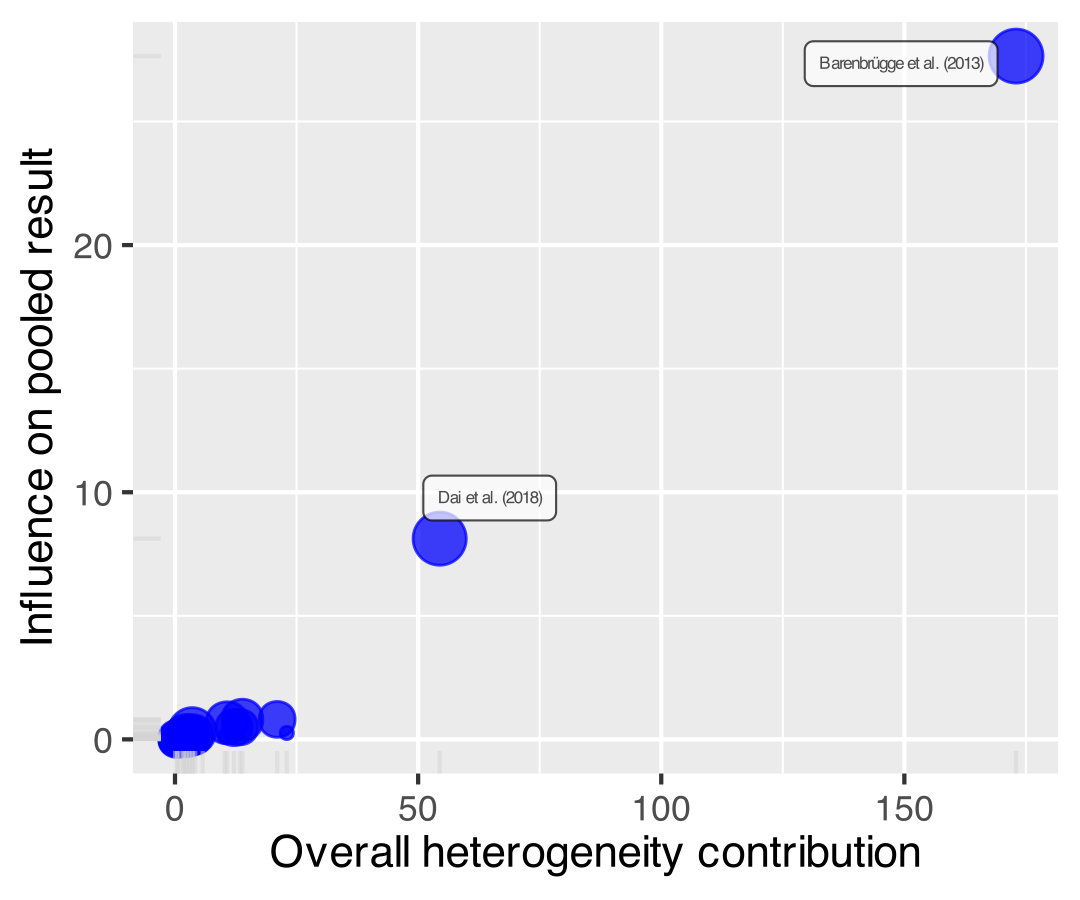  *Note.* Baujat Plot for negative metacognitions and health anxiety | |
|  | **Supplementary Figure** | | **Supplementary Figure** | |
| Safety-seeking behavior (SSB) | 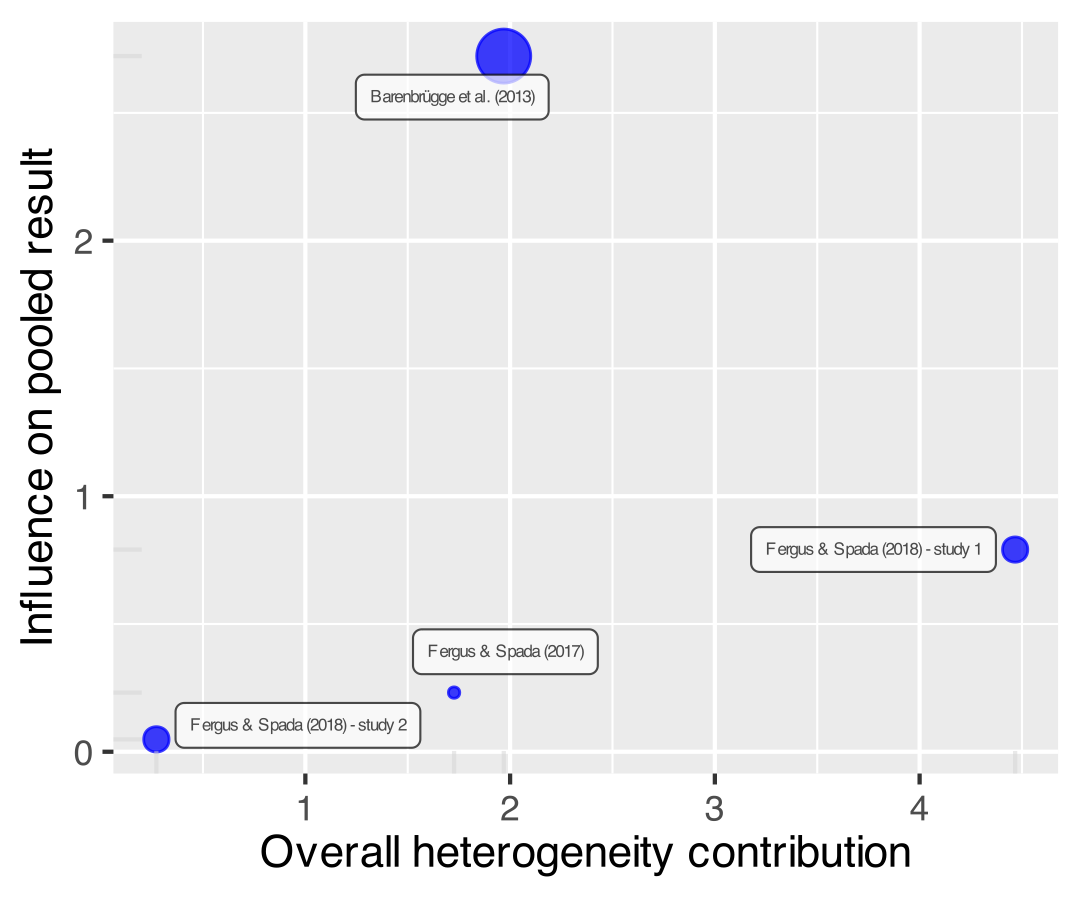  *Note.* Baujat Plot for positive metacognitions and safety-seeking behavior | | 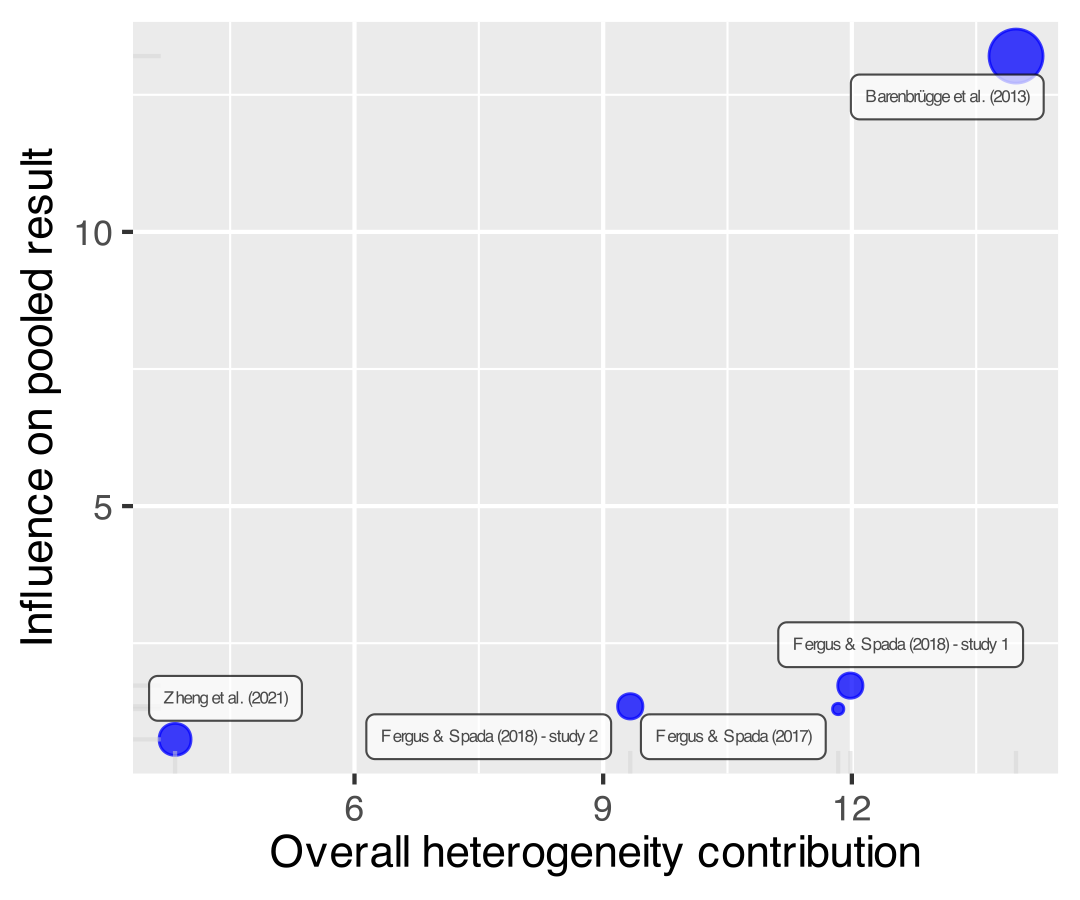  *Note.* Baujat Plot for negative metacognitions and safety-seeking behavior | |

**Leave-one-out-Plots for Metacognitions**

| **Supplementary Figure**  Positive metacognitions (PMC) | **Supplementary Figure** Negative metacognitions (NMC) |
| --- | --- |
| 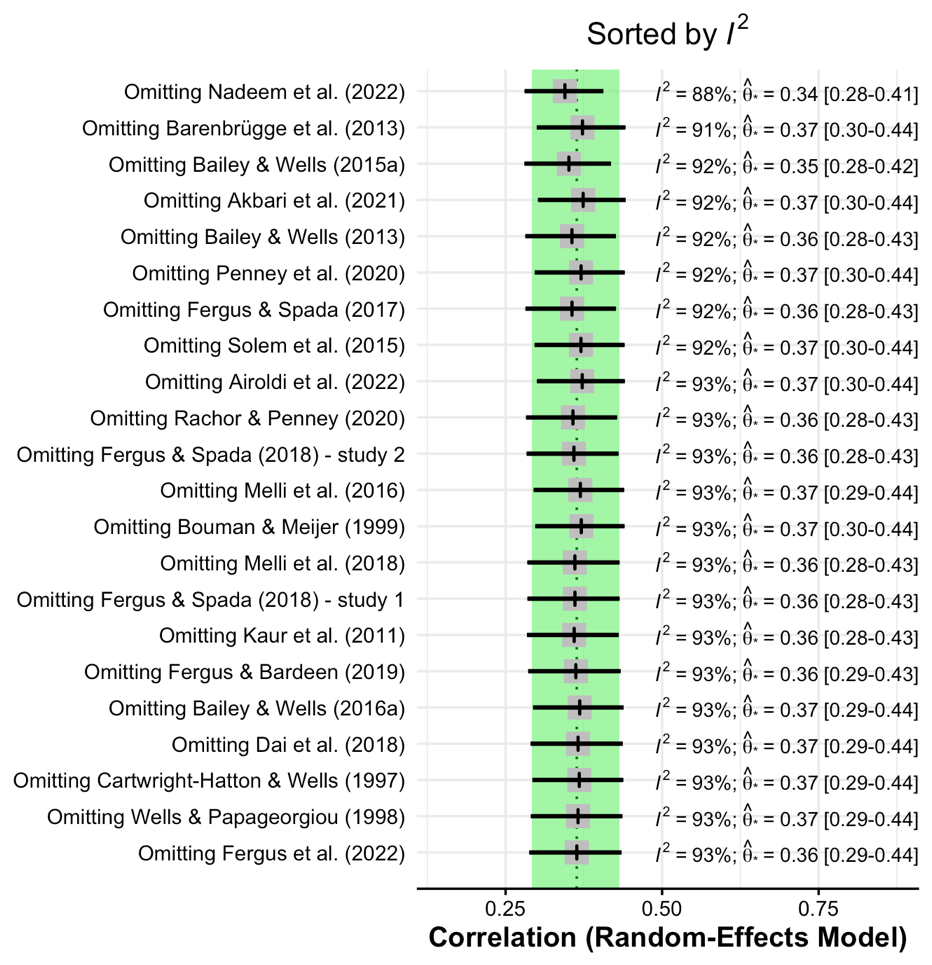 | 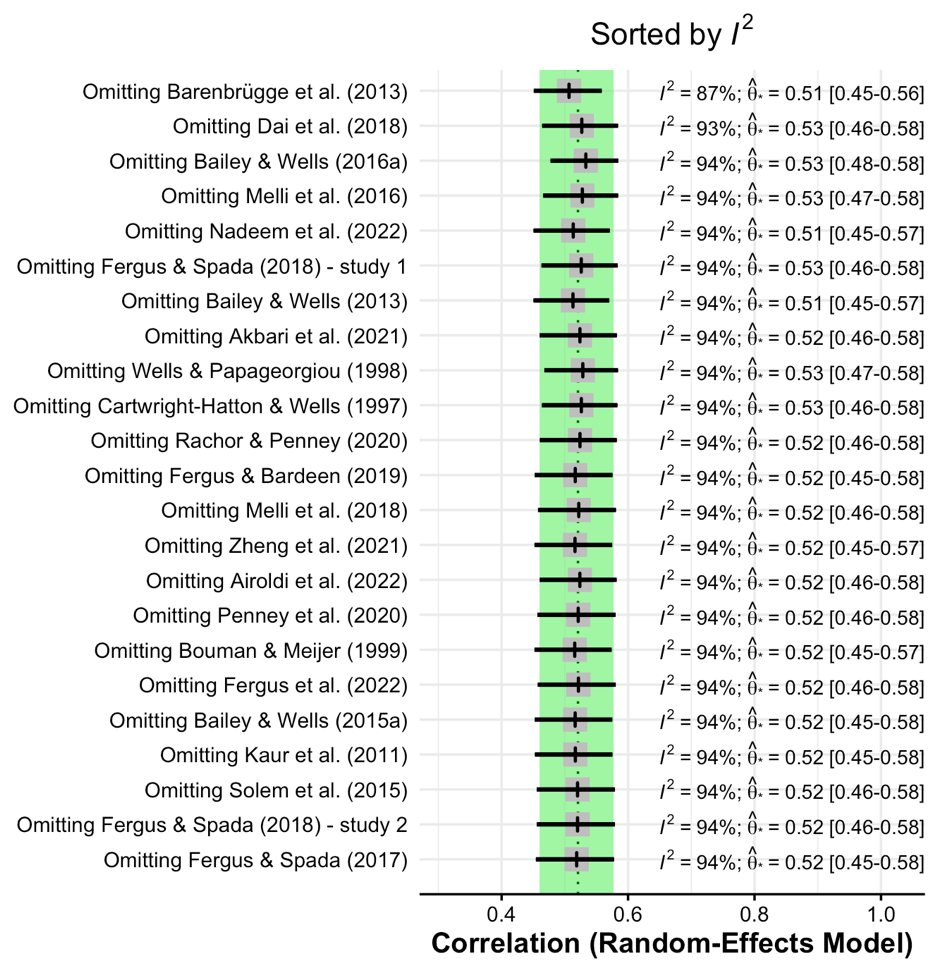 |

*Note.* Effect sizes and heterogeneity is presented leaving out one specific study, respectively.
