# Supplementary material for: Do metacognitions contribute to pathological health anxiety? A systematic review and meta-analysis
Source: PLoS One. 2025 Jul 16;20(7):e0325563. doi: 10.1371/journal.pone.0325563 (PMC12266414; doi:10.1371/journal.pone.0325563)
Supplement: S1 Fig — (DOCX) [file pone.0325563.s010.docx]

**S1 Figure. Risk of bias and Publication Bias: Contour-Enhanced Funnel Plots.**

| **Contour-Enhanced Funnel Plots for metacognitions and health anxiety** | | | |
| --- | --- | --- | --- |
| Positive metacognitions (PMC) | Negative metacognitions (NMC) |  |  |
| 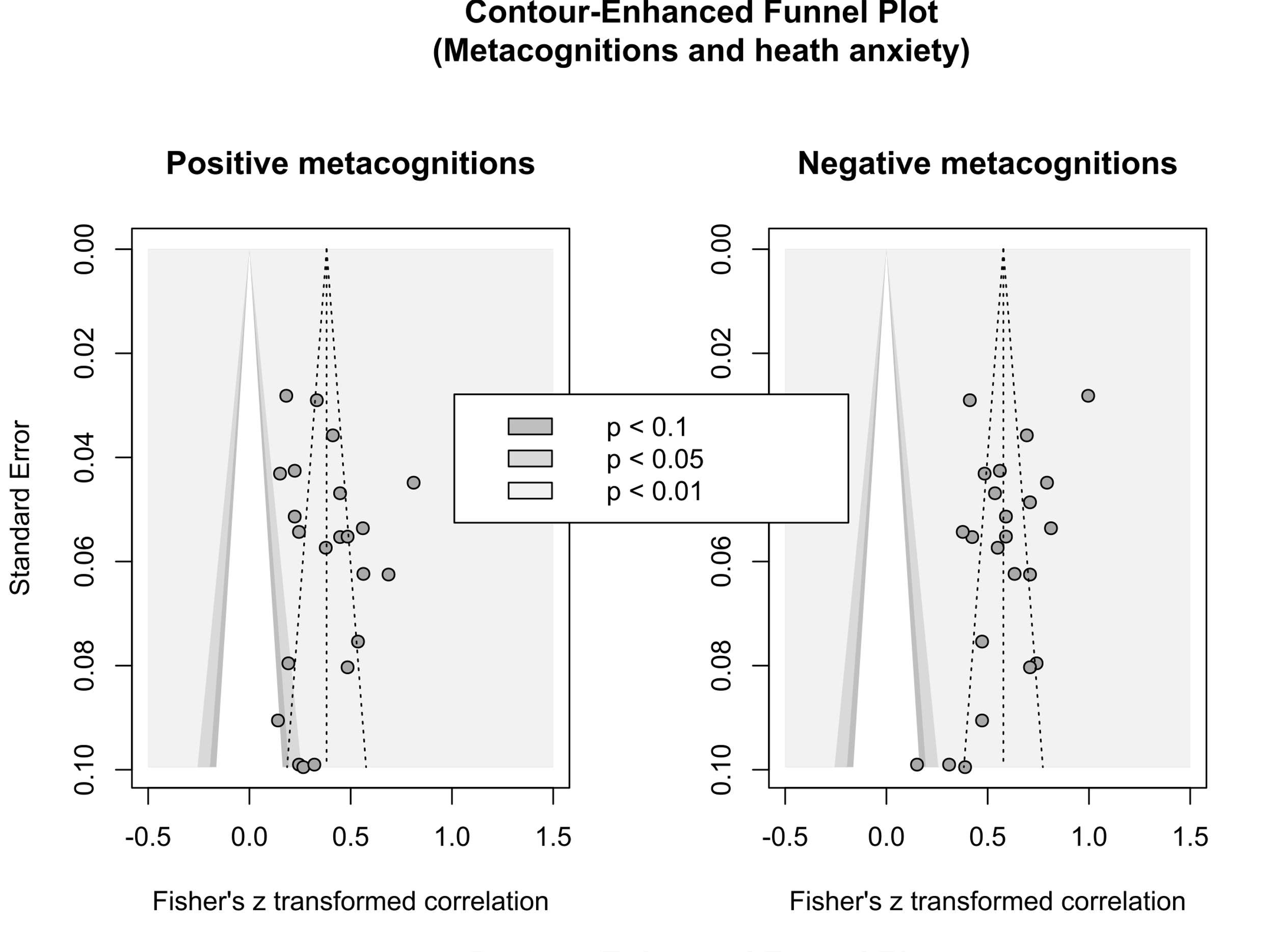 | | | |

**Contour-Enhanced Funnel Plots for metacognitions and safety-seeking behavior**

| Positive metacognitions (PMC) |
| --- |
| **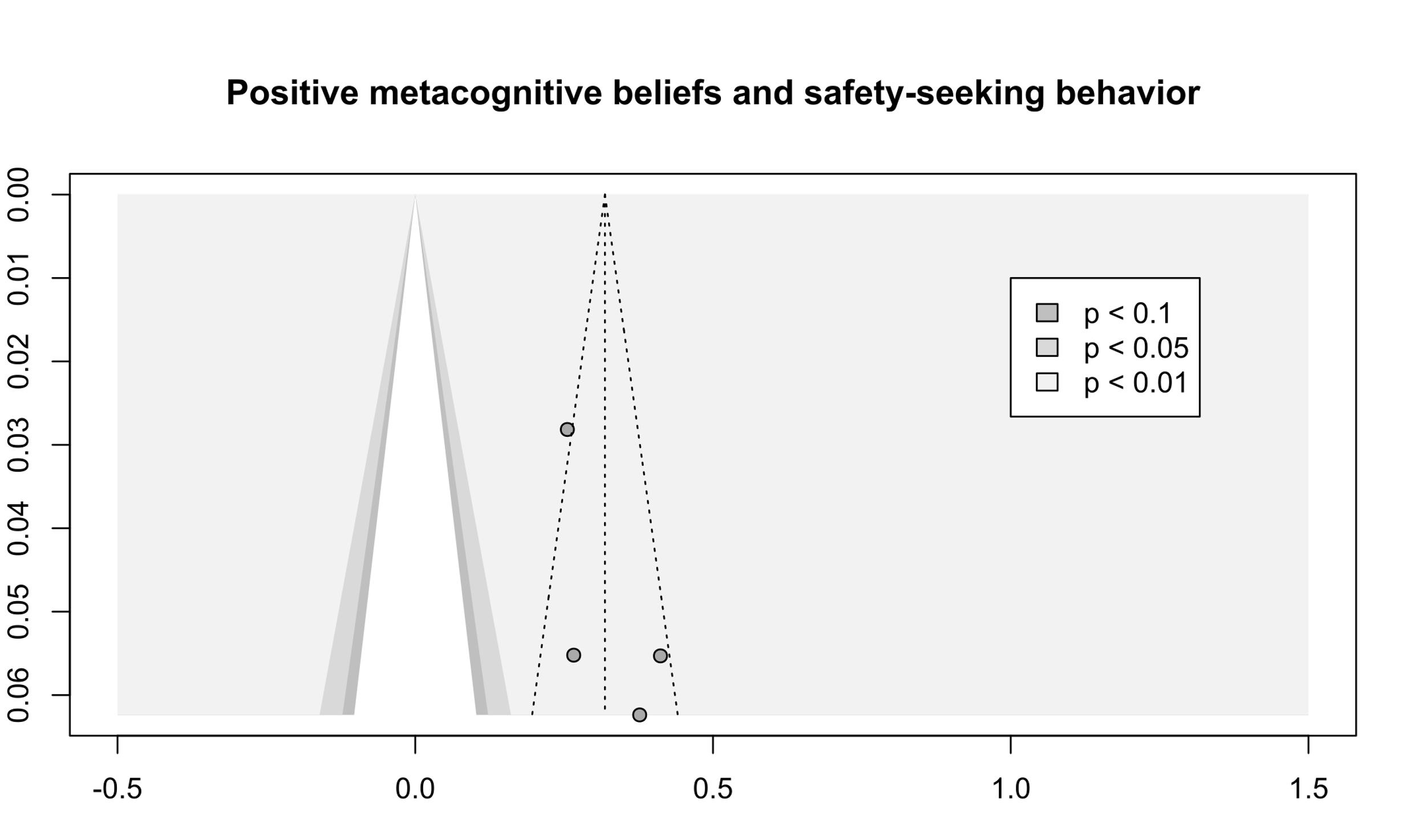** |

| Negative metacognitions (NMC) |
| --- |
| 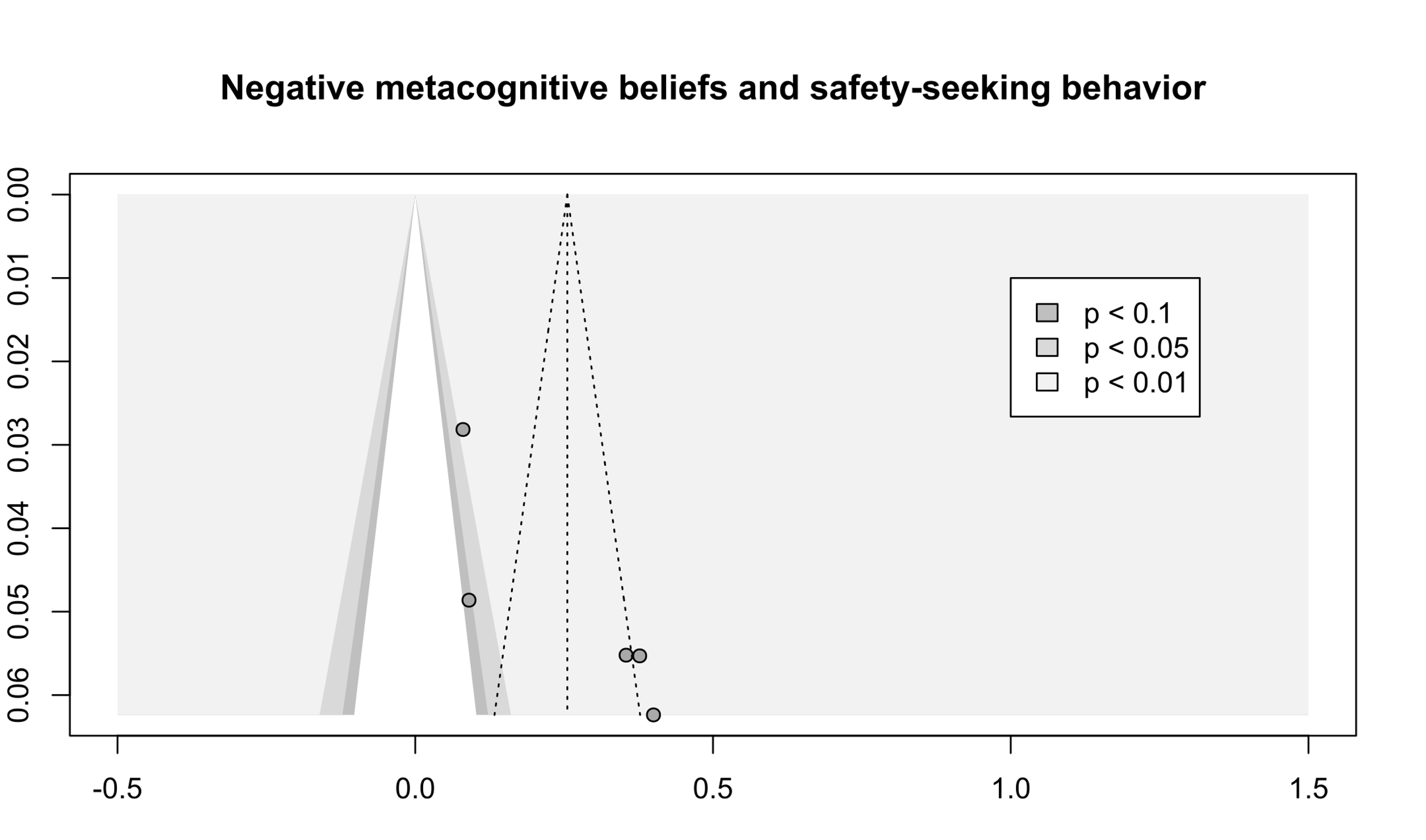 |
