# Supplementary material for: Do metacognitions contribute to pathological health anxiety? A systematic review and meta-analysis
Source: PLoS One. 2025 Jul 16;20(7):e0325563. doi: 10.1371/journal.pone.0325563 (PMC12266414; doi:10.1371/journal.pone.0325563)
Supplement: S2 Fig — (DOCX) [file pone.0325563.s011.docx]

**S2 Figure. Risk of bias and Publication Bias: *p*-Curves.**

| Positive metacognitions (PMC) and health anxiety (HA) | Negative metacognitions (NMC) and health anxiety (HA) |
| --- | --- |
| **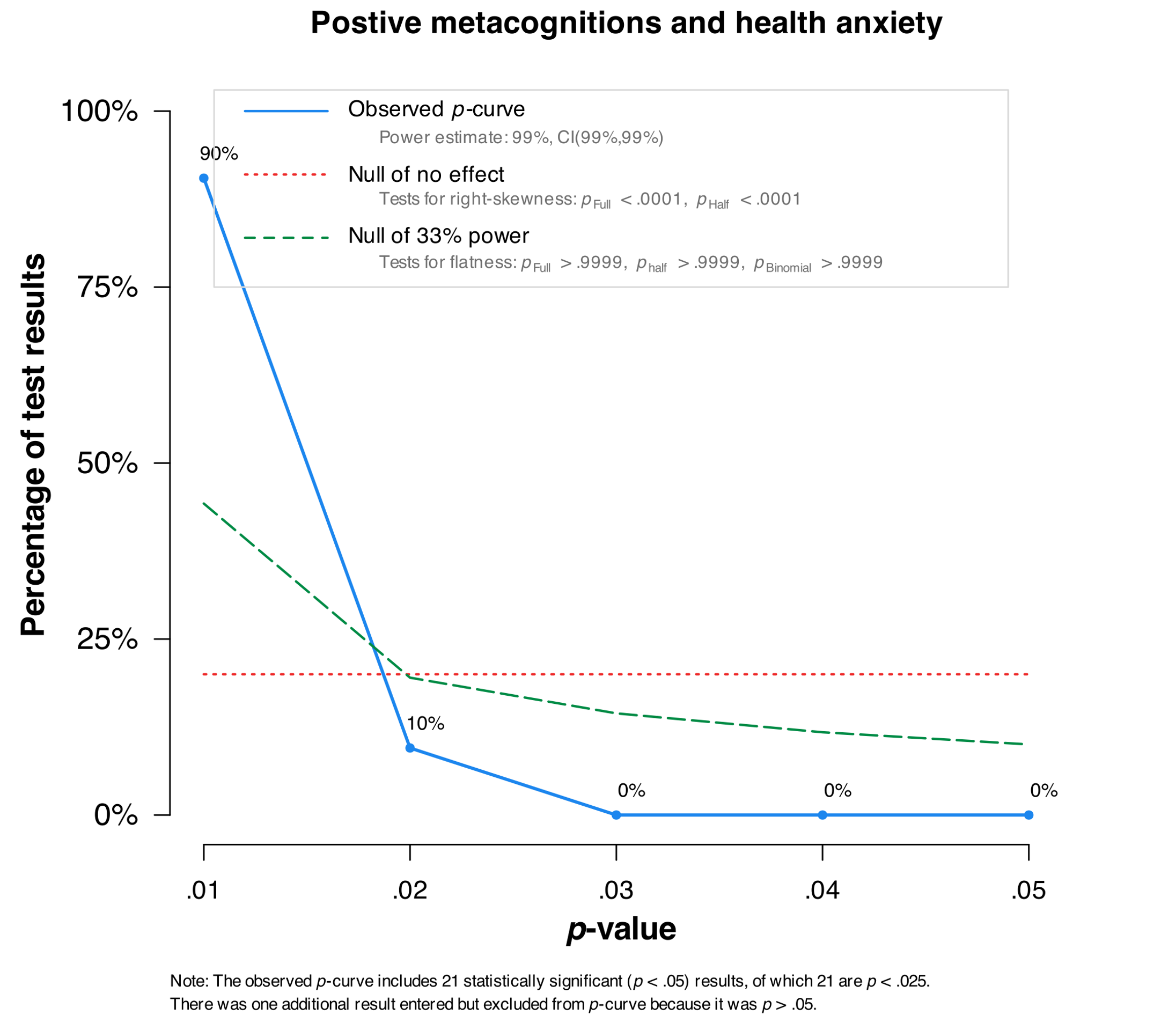** | *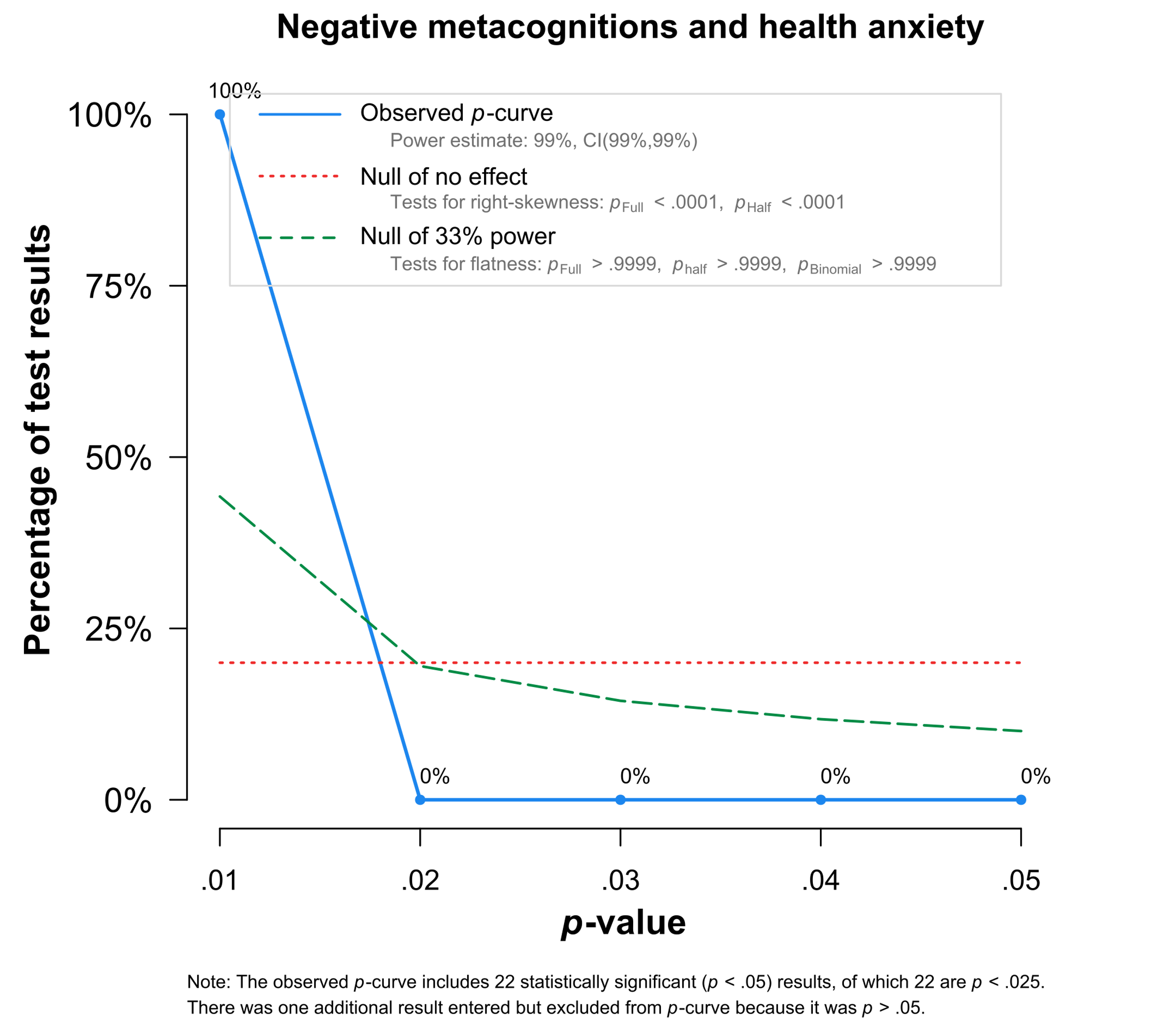* |
| *Note*. The observed *p*-curve includes 21 statistically significant (*p* < .05) results, of which 21 are *p* < .025. There was one additional result entered but excluded from *p*-curve because it was *p* > .05. | *Note*. The observed *p*-curve includes 22 statistically significant (*p* < .05) results, of which 22 are *p* < .025. There was one additional result entered but excluded from *p*-curve because it was *p* > .05. |
| Positive metacognitions (PMC) and safety-seeking behavior (SSB) | Negative metacognitions (NMC) and safety-seeking behavior (SSB) |
| *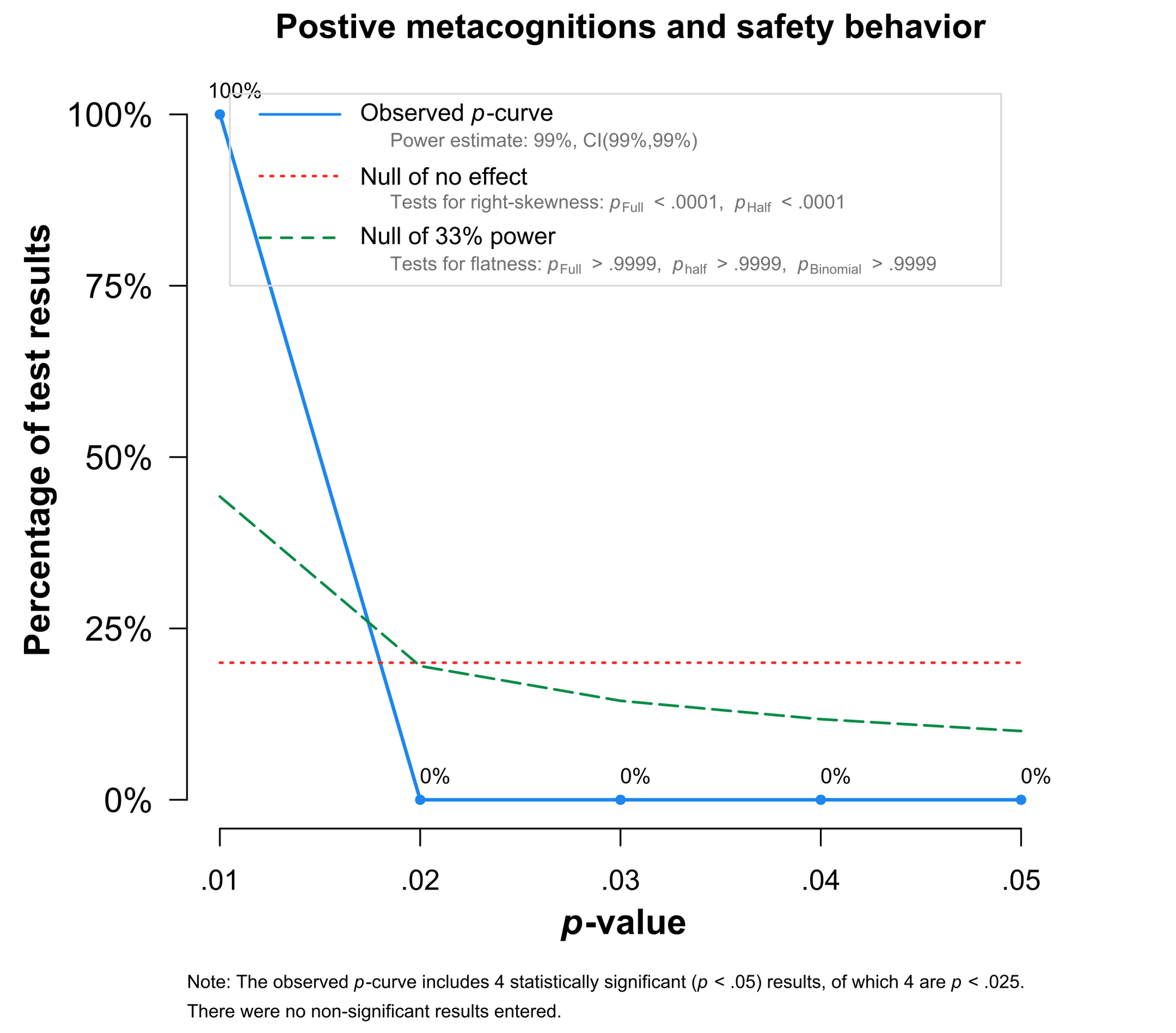* | *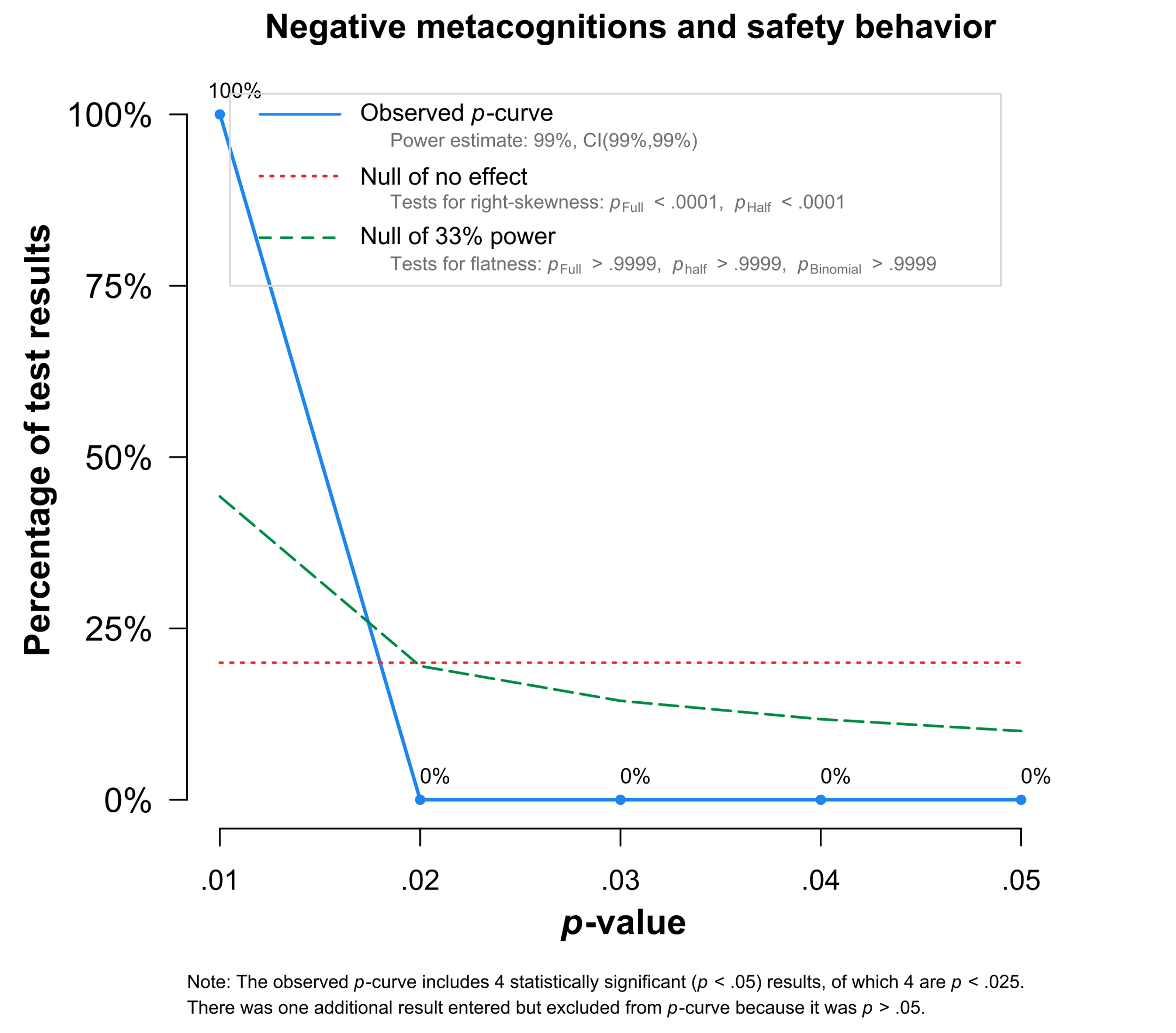* |
| *Note*. The observed *p*-curve includes 4 statistically significant (*p* < .05) results, of which 4 are *p* < .025. There were no non-significant results entered. | *Note*. The observed *p*-curve includes 4 statistically significant (*p* < .05) results, of which 4 are *p* < .025. There was one additional result entered but excluded from *p*-curve because it was *p* > .05. |
