# Supplementary material for: Do metacognitions contribute to pathological health anxiety? A systematic review and meta-analysis
Source: PLoS One. 2025 Jul 16;20(7):e0325563. doi: 10.1371/journal.pone.0325563 (PMC12266414; doi:10.1371/journal.pone.0325563)
Supplement: Attachment — Submitted filename: Response to reviewers_PLOS ONE.docx [file pone.0325563.s013.docx]

Dear Dr. Kubis, dear Reviewers,

Thank you very much for your letter dated January 30th, 2025, and for the two reviews of our manuscript titled “Do metacognitions contribute to pathological health anxiety? A systematic review and meta-analysis.” (manuscript PONE-D-24-39301R1) submitted for publication in PLOS ONE.

We thank you for the thoughtful and positive feedback and constructive suggestions for improving the manuscript. We have revised the manuscript according to your recommendations. We have carefully considered and responded in detail to each of the points made by you and the reviewers, paying particular attention to clarity and detailing information on the relevant constructs in the introduction. As a result, we believe the paper is now stronger. In the following sections, we describe in detail how we have addressed each point. References not included in the manuscript, but cited in the reply are listed below the respective comment and its reply.

**Reviewers' comments:**

**Reviewer #1:**

This article addresses the role of positive and negative metacognitions in health anxiety and their relationship with pathological safety-seeking and avoidant behaviors. Extending previous findings from other anxiety-related disorders, such as generalized anxiety disorder (GAD), this systematic review seeks to bridge a critical gap in understanding metacognitive contributions to health-related anxiety. The meta-analysis of the results of 23 studies showed positive correlations between Negative (NMC) and Positive Metacognition (PMC) on one hand and health anxiety, as well as with safe seeking behaviors. No included study assessed avoidant behavior. While the methodology appears rigorous, with clear and well-detailed inclusion criteria for the selected studies, several parts of the paper lack clarity, particularly in the introduction. Additionally, inconsistencies in formatting and figures detract from the readability of the article.

**Introduction**
The introduction is thematically relevant but requires substantial clarification.

**Comment 1.** The primary research question is unclear, and some terms central to the study, such as "health anxiety", "safety-seeking behaviors", and "avoidant behaviors" would benefit from explicit definitions to ensure accessibility to a broader readership such as the one of PLOS ONE.

**Reply:** Thank you. Our primary research aim was to provide the first meta-analysis on the relation of metacognitions and health anxiety. We were interested in specific relations of positive and negative metacognitions with health anxiety, as well as the two classes of dysfunctional behavior typical for pathological health anxiety. Based on theoretical considerations, we assumed that positive metacognitions (e.g., “I can protect myself against getting a serious disease by thinking the worst about symptoms.”) might be more related with safety seeking behaviour and negative metacognitions (e.g., “I cannot control worrying on illnesses. It is overwhelming.”) might be more related with avoidance.

We detailed the information on the terms related to health anxiety (see also our answer to your **Comment 3**). In the updated manuscript, pathological health anxiety is defined as follows: “Pathological health anxiety is diagnosed when individuals are preoccupied with having or becoming a severe disease (15) and in its severe form a significant individual and societal burden in the long-term (16).” (lines 89-91)

**Comment 2.** Although the authors cite the metacognitive and cognitive-behavioral models, these frameworks are not adequately described, making it challenging for readers unfamiliar with these concepts to contextualize the study's objectives.

**Reply:** We detailed the information on the metacognitive framework: the cognitive-attentional syndrome is explicitly described, we more clearly distinguish between positive and negative metacognitions, clarify how dysfunctional coping strategies maintain worrying and negative metacognitions and provide a more detailed account of the empirical support for the model. The first part of the introduction is rephrased as follows (text parts with relevant changes are highlighted in yellow):

“Metacognitive beliefs refer to an individual's cognitive evaluations and beliefs about their own thoughts and thinking processes (e.g., 1). Maladaptive metacognitive beliefs play a significant role in the onset and exacerbation of various mental disorders (2,3). First being established for generalized anxiety disorder (GAD), Wells metacognitive model assumes that attentional biases, perseverative thinking (i.e., worrying and ruminating) and dysfunctional coping form a cognitive-attentional syndrome that manifests in mental disorders (4,5). In contrast to traditional cognitive models, thought process regulation by metacognitive beliefs lies at the heart of this framework, while thought content is considered as subordinate (i.e., the specific worry, for example ‘What if I lose my job?’). Contradictory positive metacognitions (PMC) and negative metacognitions (NMC) are held by people with pathological worries. PMC on the helpfulness of worrying initiate perseverative thinking. As an example, a person might think that worrying helps with problem solving, motivates, prepares for eventual catastrophes, or prevents harm. Whenever a specific worry is triggered, this person will continue to worry, because of those PMC. Engagement in perseverative thinking then activates NMC about the controllability and harmfulness of worries. When thoughts are experienced as particularly uncontrollable, people tend to engage in dysfunctional coping strategies, e.g., try to suppress their thoughts, or try to avoid triggers. As a result, they are more likely to remain stuck in perseverative thinking processes than people who attribute less importance to their thoughts. In other words, those strategies rather reinforce worrying and the perception of uncontrollability of worries (i.e., NMC). Various studies demonstrated that especially negative metacognitive beliefs relate to pathological worry in the context of GAD (5–8) and predict the duration, intensity, and burdensomeness of subsequent worry episodes (9,10), as well as generalized anxiety longitudinally (2).

In addition to their relevance for GAD, metacognitions are supposed to specifically impact mental disorders that are characterized by any kind of intrusive thoughts (11). In line with this, besides GAD, especially large effect sizes of NMC were found for obsessive compulsive disorder and major depression when contrasting different psychopathologies meta-analytically (2). Moreover, metacognitive therapy has been proven to be an effective transdiagnostic therapy, superior to standard cognitive behavioral therapy (for a meta-analysis see 12).” (lines 55-84)

**Comment 3.** The relationship between health anxiety, safety-seeking behaviors, and avoidance behaviors is not sufficiently detailed, leaving the reader without a clear understanding of how these constructs are conceptualized or interrelated.

**Reply:** Thank you for this important remark. We detailed the information on dysfunctional behaviors in illness anxiety as follows:

“Due to uncertainty concerning their health status, individuals may engage in excessive information sampling (e.g., checking the body, reassurance seeking, googling). Conversely, they might avoid any information that triggers their health anxiety (e.g., conversations about medical conditions, medical examinations, thinking about being ill). Safety seeking behaviors and avoidant behaviors are the two classes of dysfunctional behaviors typically maintaining pathological health anxiety. According to the *Diagnostic and Statistical Manual of Mental Disorders* (DSM 5), individuals can be categorized as either care-seeking or care-avoidant (15). However, most patients engage in both dysfunctional behaviors, and intra- and interindividual fluctuations remain poorly understood (17).” (lines 91-100)

**Methods**
The methodology is robust, and the explanation of study selection and bias assessment is clear. However, some areas might require additional detail:

**Comment 4.** How many studies reported regression coefficients and how were they transformed into correlation coefficients?

**Reply:** Only Bailey and Wells (2016) report regression coefficients. The three betas reported in this study were transformed into correlation coefficients using the esc_beta function (see uploaded Excel sheet and R script ‘2_RScript_MC-IA_datapreparation’). We have adjusted information in the manuscript as follows:

„Regression coefficients instead of correlation coefficients were reported in one study (33). The three extracted standardized regression coefficients were transformed into correlation coefficients with the ‘esc_beta’ function in R.“ (lines 203-205)

**Comment 5.** The section on data preparation mentions subgroups, what do they refer to?

**Reply**: Three of the studies (Barenbrügge et al., 2013; Bouman & Meijer, 1999; Fergus et al., 2022) report relevant correlation coefficients for different subgroups. Barenbrügge et al. (2013) distinguish between participants in ‘good health’ vs. in ‘poor health’ (whereby poor health was indicated by a self-reported doctor-diagnosed physical illness in the last three months, or one that persisted longer than the last three months). Bouman & Meijer (1999) categorize the sample into ‘hypochondriacal patients’, ‘normal controls’ and ‘psychology students’. And Fergus et al. (2022) differentiate between ‘non-latinx-black’, ‘latinx’ and ‘non-latinx-white’ subgroups. Information on the different subgroups as well as the corresponding correlation coefficients can be found in the uploaded Excel sheet. We provide one example for the subgroups in the section on data preparation and refer to the uploaded data and scripts as follows:

In the following citation, changed passages are highlighted in yellow: “For studies which used an instrument with more than one relevant subscale, or which reported the correlations separately for subgroups (*k* = 3, e.g., participants in good vs. poor health, 20), the corresponding correlation coefficients were averaged using Fisher’s *z*-transformation and weighted based on sample sizes (see uploaded data and scripts).“ (lines 205-209)

**Comment 6.** Figure 2 should include titles for panels A and B to improve interpretability.

**Reply:** In the title of Figure 2, we refer to both panels as follows: “**Fig 2. Forest Plots for the relation of health anxiety and positive (A) as well as negative metacognitive beliefs (B).**” We assume that the interpretability is clear once the manuscript has been set, the caption is placed directly above the Figure.

**Comment 7.** For analogue samples, it might be helpful to report the average levels and variability in health anxiety.

**Reply:** Thank you for this remark. We extracted reported levels of health anxiety for each study. We summarize the findings in the sample characteristics section:

“The average level of health anxiety varied between the studies ranging from low to severely health anxious on the respective self-report scales. Often, averaged health anxiety was mild. All mean values and standard deviations for the included studies can be found in the supplementary Table S5.” (lines 289-293)

and provide the detailed information in the supplementary Table S5.

**S5 Table. Sample characteristics.**

*Average health anxiety scores of the included studies*

| Study | | *N* | Question-naire | Possible range of scores in the respective question-naires | Average Health Anxiety Score *M* (*SD*) | Interpretation of Averaged Health Anxiety Score | Sample |  |
| --- | --- | --- | --- | --- | --- | --- | --- | --- |
|  | |  |  |  |  |  |  | |
| Melli et al. 2018 (1) | | 458 | HAQ | 21 to 84 | 64.69 (10.78) | - | clinical | |
| Melli et al. 2016 (2) | | 342 | HAQ | 21 to 84 | 32.47 (8.70) | - | analogue | |
| Airoldi et al. 2022 (3) | | 125 | SHAI-14 | 0 to 42 | 25.69 (6.08) | above cut-off, mild | analogue | |
| Nadeem et al. 2022 (4) | | 500 | SHAI-14 | 0 to 42 | 18.72 (8.77) | above cut-off, mild | analogue | |
| Akbari et al. 2022 (5) | | 541 | SHAI | 0 to 54 | 29.74 (6.13) | above cut-off | analogue | |
| Dai et al. 2018 (6) | | 1191 | SHAI | 0 to 54 | 11.73 (5.32) | below cut-off | analogue | |
| Penney et al. 2020 (7) | | 565 | SHAI | 0 to 54 | 16.47 (8.18) | above cut-off | analogue | |
| Rachor & Penney 2020 (8) | | 179 | SHAI | 0 to 54 | 20.27 (7.46) | above cut-off | analogue | |
| Bailey & Wells 2016 (9) | | 105 | WI (Likert) | 14 to 70 | 26.36 (9.36) | below cut-off | analogue | |
| Bailey & Wells 2013 (10) | | 351 | WI (Likert) | 14 to 70 | 26.37 (10.14) | below cut-off | analogue | |
| Bailey & Wells 2015 (11) | | 235 | WI (Likert) | 14 to 70 | not reported |  | analogue | |
| Barenbrügge et al. 2013 (12) | | 1264 | WI (dichotome) | 0 to 14 | 3.39 (2.87) | below cut-off | analogue | |
| Bouman & Meijer 1999 (13) | |  |  |  |  |  |  | |
|  | normal controls | 25 | WI (dichotome) | 0 to 14 | 2.0 (2.0) | below cut-off | analogue | |
|  | psychology students | 122 | WI (dichotome) | 0 to 14 | 2.7 (2.2) | below cut-off | analogue | |
|  | hypochondrial patients | 14 | WI (dichotome) | 0 to 14 | 10.9 (2.2) | above cut-off | clinical | |
| Kaur et al. 2011 (14) | | 158 | WI (Likert) | 14 to 70 | 13.94 (9.00) | below cut-off | analogue | |
| Solem et al. 2015 (15) | | 382 | WI (Likert) | 14 to 70 | 23.3 (7.70) | below cut-off | analogue | |
| Fergus & Bardeen 2019 (16) | | 785 | WI-6 | 6 to 30 | not reported | - | analogue | |
| Fergus & Spada 2017 (17) | | 337 | WI-6 | 6 to 30 | 13.14 (7.68) | - | analogue | |
| Fergus & Spada 2018 (18) | |  |  |  |  |  |  | |
|  | Study 1 | 330 | WI-6 | 6 to 30 | 13.66 (5.20) | - | analogue | |
|  | Study 2 | 331 | WI-6 | 6 to 30 | 13.7 (5.34) | - | analogue | |
| Fergus et al. 2022 (19) | |  |  |  |  |  |  | |
|  | non-latinx black | 123 | WI-6 | 6 to 30 | 14.53 (5.95) | - | analogue | |
|  | latinx | 104 | WI-6 | 6 to 30 | 14.53 (5.46) | - | analogue | |
|  | non-latinx white | 80 | WI-6 | 6 to 30 | 15.71 (6.72) | - | analogue | |
| Zheng et al. 2021 (20) | | 426 | WI-6 | 6 to 30 | not reported | - | analogue | |
| Cartwright-Hatton & Wells 1997 (21) | | 47 | AnTI | 6 to 24 | 9.5 (3.8) | - | analogue | |
| Wells & Papageorgiou 1998 (22) | | 105 | AnTI | 6 to 24 | 9.4 (not reported) | - | analogue | |

Abbreviations: HAQ = Health Anxiety Questionnaire, *note* response format of the Italian version used differs from the English version (shifting of the scale values), determined by personal correspondence with Gabriele Melli, Melli et al. (29) report average values of the Italian HAQ version of: *M* = 31.6, *SD* = 7.9 for men and *M* = 36, *SD* = 10.6 for women, HAI-14 = Short Health Anxiety Inventory-14 (14 Items), *note* a cut-off of 18 was suggested for analogue samples (‘healthy context’) and values are interpreted as follows: 0-27: no to mild health anxiety, 28-32: moderate health anxiety, 33-42: substantial health anxiety (23), SHAI = Short Healthy Anxiety Inventory (18 Items), *note* a cut-off of > 14 is interpreted as noticeable health anxious (24), WI = Whiteley Index (14 Items), *note* the cut-off depends on the response format (dichotome vs. 5-point Likert-scale), for WI (dichotome) a cut-off of 5 was used to discriminate persons with severe health anxiety from those without severe health anxiety (25) and for WI (Likert) a cut off score of 40 or above indicates the presence of hypochondriasis (26), WI-6 = Whiteley Index (6 Items), *note* Fergus et al. (27) report *M* = 21.28, *SD* = 5.54 for severe health anxiety and *M* = 13.49, *SD* = 5.60 for no severe health anxiety, there is no cut-off criterion, AnTI = Anxious Thought Inventory: the subscale health worry was used to determine the level of health anxiety, *note* Vázque Morejón et al. (28) report *M* = 12.66, *SD* = 4.84 in a clinical sample

**References**

1.	Melli G, Bailey R, Carraresi C, Poli A. Metacognitive beliefs as a predictor of health anxiety in a self‐reporting Italian clinical sample. Clin Psychol Psychother. 2018 Mar;25(2):263–71.

2.	Melli G, Carraresi C, Poli A, Bailey R. The role of metacognitive beliefs in health anxiety. Personal Individ Differ. 2016 Jan 1;89:80–5.

3.	Airoldi S, Kolubinski DC, Nikčević AV, Spada MM. The relative contribution of health cognitions and metacognitions about health anxiety to cyberchondria: A prospective study. J Clin Psychol. 2022 May;78(5):809–20.

4.	Nadeem F, Malik N, Atta M, Ullah I, Martinotti G, Pettorruso M, et al. Relationship between Health-Anxiety and Cyberchondria: Role of Metacognitive Beliefs. J Clin Med. 2022 May 5;11(9):2590.

5.	Akbari M, Spada MM, Nikčević AV, Zamani E. The relationship between fear of COVID‐19 and health anxiety among families with COVID‐19 infected: The mediating role of metacognitions, intolerance of uncertainty and emotion regulation. Clin Psychol Psychother. 2021 Nov;28(6):1354–66.

6.	Dai L, Bailey R, Deng Y. The reliability and validity of the Chinese version of the Metacognitions about Health Questionnaire in college students. Qual Life Res. 2018 Apr 1;27(4):1099–108.

7.	Penney AM, Rachor GS, Deleurme KA. Differentiating the roles of intolerance of uncertainty and negative beliefs about worry across emotional disorders. J Exp Psychopathol. 2020 Oct 1;11(4):204380872097007.

8.	Rachor GS, Penney AM. Exploring metacognitions in health anxiety and chronic pain: a cross-sectional survey. BMC Psychol. 2020 Dec;8(1):81.

9.	Bailey R, Wells A. The contribution of metacognitive beliefs and dysfunctional illness beliefs in predicting health anxiety: An evaluation of the metacognitive versus the cognitive models. Clin Psychol. 2016 Nov 1;20(3):129–37.

10.	Bailey R, Wells A. Does Metacognition Make a Unique Contribution to Health Anxiety When Controlling for Neuroticism, Illness Cognition, and Somatosensory Amplification? J Cogn Psychother. 2013;27(4):327–37.

11.	Bailey R, Wells A. Metacognitive beliefs moderate the relationship between catastrophic misinterpretation and health anxiety. J Anxiety Disord. 2015 Aug 1;34:8–14.

12.	Barenbrügge J, Glöckner-Rist A, Rist F. Positive und negative Metakognitionen über Krankheitssorgen. Psychotherapeut. 2013 Nov;58(6):560–8.

13.	Bouman TK, Meijer KJ. A preliminary study of worry and metacognitions in hypochondriasis. Clin Psychol Psychother. 1999;6(2):96–101.

14.	Kaur A, Butow P, Thewes B. Do Metacognitions Predict Attentional Bias in Health Anxiety? Cogn Ther Res. 2011 Dec;35(6):575–80.

15.	Solem S, Borgejordet S, Haseth S, Hansen B, Håland Å, Bailey R. Symptoms of health anxiety in obsessive–compulsive disorder: Relationship with treatment outcome and metacognition. J Obsessive-Compuls Relat Disord. 2015 Apr;5:76–81.

16.	Fergus TA, Bardeen JR. The Metacognitions Questionnaire–30: An Examination of a Bifactor Model and Measurement Invariance Among Men and Women in a Community Sample. Assessment. 2019 Mar;26(2):223–34.

17.	Fergus TA, Spada MM. Cyberchondria: Examining relations with problematic Internet use and metacognitive beliefs. Clin Psychol Psychother. 2017 Nov;24(6):1322–30.

18.	Fergus TA, Spada MM. Moving toward a metacognitive conceptualization of cyberchondria: Examining the contribution of metacognitive beliefs, beliefs about rituals, and stop signals. J Anxiety Disord. 2018 Dec;60:11–9.

19.	Fergus TA, Wilder K, Koester P, Kelley LP, Griggs JO. Metacognitive beliefs about uncontrollability relate most strongly to health anxiety among US‐based non‐Latinx White primary care patients: Comparing strength of relations with US‐based non‐Latinx Black and Latinx primary care patients. Clin Psychol Psychother. 2022 Jul;29(4):1331–41.

20.	Zheng H, Kyung Kim H, Joanna Sin SC, Theng YL. A theoretical model of cyberchondria development: Antecedents and intermediate processes. Telemat Inform. 2021 Oct;63:101659.

21.	Cartwright-Hatton S, Wells A. Beliefs about Worry and Intrusions: The Meta-Cognitions Questionnaire and its Correlates. J Anxiety Disord. 1997 May;11(3):279–96.

22.	Wells A, Papageorgiou C. Relationships between worry, obsessive–compulsive symptoms and meta-cognitive beliefs. Behav Res Ther. 1998 Sep;36(9):899–913.

23.	Österman S, Axelsson E, Lindefors N, Hedman-Lagerlöf E, Hedman-Lagerlöf M, Kern D, et al. The 14-item short health anxiety inventory (SHAI-14) used as a screening tool: appropriate interpretation and diagnostic accuracy of the Swedish version. BMC Psychiatry. 2022 Nov 14;22(1):701.

24.	Rode S, Salkovskis P, Dowd H, Hanna M. Health anxiety levels in chronic pain clinic attenders. J Psychosom Res. 2006 Feb;60(2):155–61.

25.	Hedman E, Lekander M, Ljótsson B, Lindefors N, Rück C, Andersson G, et al. Optimal Cut-Off Points on the Health Anxiety Inventory, Illness Attitude Scales and Whiteley Index to Identify Severe Health Anxiety. Elhai JD, editor. PLOS ONE. 2015 Apr 7;10(4):e0123412.

26.	Gerdes TT, Noyes R, Kathol RG, Phillips BM, Fisher MM, Morcuende MA, et al. Physician recognition of hypochondriacal patients. Gen Hosp Psychiatry. 1996 Mar;18(2):106–12.

27.	Fergus TA, Kelley LP, Griggs JO. Examining the Whiteley Index-6 as a screener for DSM-5 presentations of severe health anxiety in primary care. J Psychosom Res. 2019 Dec;127:109839.

28.	Vázquez Morejón AJ, Vázquez-Morejón R, Muñoz-Fernández N. Anxious Thoughts Inventory (AnTI). Psychometric characteristics of an adaptation to the Spanish clinical population. Ansiedad Estrés. 2020 Jul;26(2–3):91–7.

29.	Melli G, Coradeschi D, Smurra R. La versione italiana dell’Health Anxiety Questionnaire: Attendibilità e struttura fattoriale [The Italian version of Health Anxiety Questionnaire: Reliability and factorial analysis]. Psicoter Cogn E Comportamentale. 2007;13(1):37–48.

**Results**
The results section provides useful information but requires clarification in several places:

**Comment 8.** In the study selection paragraph, it is unclear whether the cited sample sizes (n) are among the 23 included studies. While Figure 1 and Table 1 are clear and helpful, ensuring that the text aligns with these visuals would improve consistency.

**Reply:** Thank you for bringing this to our attention. We adapted the paragraph, ensuring the text and the visuals are now consistent. To make it clear that the final sample of studies is 23, we changed the order of the information concerning sample sizes and added an additional sentence at the beginning. The adapted paragraph reads as follows:

In the following citation, changed passages are highlighted in yellow: “After the title-abstract screening, there were 51 studies sought for retrieval. Having access to 48 abstracts only, the authors of 3 studies were contacted to provide their full-texts. One study was still under review, another study was never published and the authors of the third study did not answer. There were 14 full-texts with missing or incomplete data reports. The authors of 5 studies sent us the relevant information. Concerning the other studies, the data were not accessible anymore (*n* = 4) or the researchers did not reply (*n* = 5). Finally, a total of 23 published studies were included in the meta-analysis. The PRISMA flow diagram (39) is shown in Fig 1. For an overview of the included studies see Table 1. Studies that were included in the meta-analysis but are not discussed in the paper can be found in the supplement (S4).“ (lines 249-257)

**Comment 9.** The introduction suggests a causal relationship and sequentiality between PMC and NMC, was the correlation between the two evaluated?

**Reply:** In the theoretical framework, Wells assumes indeed that the process of worrying is initiated by positive metacognitions, which in turn leads to negative metacognitions. This actually implies a causal relationship and a specific sequence in the development of pathological worries. As causal relationships must be investigated experimentally and longitudinally, such proof of concepts studies is rare up to now (e.g., Nordahl et al., 2023). To the best of our knowledge no longitudinal study on metacognitions in **health anxiety** exists and experimental manipulations of metacognitions are also lacking (for an example of experimental manipulation of metacognitions in generalized anxiety see Andor et al., 2008). We felt it was a very good first step to summarize how strongly metacognitions are related to health anxiety without considering causality in this meta-analysis. Aligned with the preregistration we focused on the associations between either PMC or NMC with HA and dysfunctional behaviors typical for health anxiety. The correlation between PMC and NMC was not specifically evaluated.

Nordahl H, Anyan F, Hjemdal O. Prospective relations between dysfunctional metacognitive beliefs, metacognitive strategies, and anxiety: Results from a four-wave longitudinal mediation model. Behav Ther. 2023;54(5):765-776. <https://doi.org/10.1016/j.beth.2023.02.003>.

**Comment 10.** A subgroup analysis based on gender (female/male) could provide additional insights, given the potential for gender differences in health anxiety and related behaviors.

**Reply:** We did not consider gender differences in our research questions and therefore did not preregister such subgroup analysis. To our knowledge, there are currently no clear indications of gender differences in health anxiety (for a review see Weck et al., 2014; Bailer et al., 2013) or metacognitions in the literature (Barenbrügge et al., 2013; Lenzo et al., 2016). Furthermore, only a few of the included studies in our meta-analysis report gender-specific data for health anxiety or related behaviors (Akbari et al., 2022; Bailey & Wells, 2013; Dai et al., 2018; Zheng et al., 2021). In these cases, gender was often included as a control variable and had no significant effect on any construct of interest. Barenbrügge et al. (2013) included gender as a covariate in their confirmatory factor analysis on their metacognitions about health anxiety questionnaire and found no effect of gender on reported levels of positive or negative metacognitions. Fergus and Bardeen (2019) report an adequate model fit for the bifactorial model of the MCQ-30 among men and women. Only Solem et al. (2015) report that gender was a significant predictor of health anxiety in a regression analysis. They conclude that women have higher scores on the WI than men. We decided to not add a subgroup analysis based on gender.

Weck F, Richtberg S, Neng JMB. Epidemiology of hypochondriasis and health anxiety: Comparison of different diagnostic criteria. Curr Psychiatry Rev. 2014;10(1):14-23. doi: 10.2174/1573400509666131119004444.

Lenzo V, Toffle ME, Tripodi F, Quattropani MC. Gender differences in anxiety, depression and metacognition. In: Bekirogullari Z, Minas MY, Thambusamy RX, editors. Clinical & Counselling Psychology - CPSYC 2016. European Proceedings of Social and Behavioural Sciences. 2016;9:1-16. doi: 10.15405/epsbs.2016.05.02.1.

**Discussion**
The discussion draws meaningful parallels with findings from related pathologies, such as GAD and obsessive-compulsive disorder (OCD), but lacks specificity regarding health anxiety.

**Comment 11.** What are the specificities of health anxiety compared to the other cited pathologies? Exploring such differences could highlight the unique contributions of metacognitive processes in this context.

**Reply:** Thank you for this remark. Comparable to previous reviews, we assume that negative metacognitions can be considered a transdiagnostic factor with high explanatory power for psychopathology in general. The effect of positive metacognitions is more heterogeneous across syndromes and might be more important for health anxiety compared to GAD and OCD (see our discussion on PMC lines 424 to 435). However, direct comparisons of clinical samples with different diagnoses and studies, which link metacognitions to specific dysfunctional coping mechanisms are needed to elucidate the potentially specific role of PMC for health anxiety. We decided to add a sentence to the ‘Future directions’ section, highlighted in yellow:

“Although cognitive behavioral therapy is effective for treating pathological health anxiety, response and remission rates indicate that 50% of affected individuals do not profit to a satisfactory extent (74). This meta-analysis provides evidence for Wells model, according to which people with increased anxiety hold both, PMC and contradictory NMC (4). Thus, it seems plausible to include metacognitions in case conceptualizations. However, the interplay between these metacognitions and dysfunctional coping strategies has not yet been sufficiently investigated in illness anxiety. Therefore, it seems sensible to attempt to better understand the mechanisms involved in the emergence and perpetuation of health anxiety. To increase our understanding, future studies should investigate clinical samples, manipulate metacognitions (e.g., 6) and examine their influence on SSB and AB. The potentially specific role of PMC for health anxiety and SSB has to be proven in studies comparing different clinical samples and studies which link specific metacognitions to different dysfunctional strategies (SSB, avoidance, hypervigilance). Finally, it would be of interest to examine, whether metacognitions explain incremental variance beyond and above other mechanisms relevant for pathological health anxiety (75), explain SSB and AB in day to day life and predict health anxiety longitudinally.“ (lines 453-468)

**Comment 12.** Were the subfacets of health anxiety questionnaires analyzed in the present study?

**Reply:** No, in our analyses we only considered the total score for health anxiety. Our focus was on metacognitions and the behavioral component of health anxiety, the two types of safety behaviors. Analyzing also the subfacets of health anxiety was beyond the scope of our preregistered research hypotheses. Furthermore, the number and type of subscales differ depending on the questionnaire used (see the following Table).

| **Table. Questionnaires used to assess health anxiety and the corresponding subscales.** | |
| --- | --- |
| Questionnaire | Subscales |
| WI | (1) Disease conviction (2) Disease phobia (3) Bodily preoccupation |
| WI-6 | - |
| SHAI | (1) The feared likelihood of becoming ill (2) The feared negative consequences of becoming ill |
| SHAI-14 | - |
| HCQ | (1) Likelihood of contracting or having an illness (2) Awfulness of illness (3) Inability to cope with illness (4) Inadequacy of medical services for treating illness |
| AnTI* | (1) Social worry (2) Health worry (3) Meta worry |
| HAQ | (1) Health worry and preoccupation (2) Fear of illness and death (3) Reassurance-seeking behavior (4) Interference with life |
| **Note*. We only included the health worry subscale of this inventory. | |

**Comment 13.** The authors cite Wells' metacognitive model in the introduction, how do the findings relate to this model? A more explicit connection would improve the theoretical grounding of the results.

**Reply:** Thank you. We now link our findings more explicitly to Wells’ metacognitive model in the Future directions section:

“Although cognitive behavioral therapy is effective for treating pathological health anxiety, response and remission rates indicate that 50% of affected individuals do not profit to a satisfactory extent (74). This meta-analysis provides evidence for Wells model, according to which people with increased anxiety hold both, PMC and contradictory NMC (4). Thus, it seems plausible to include metacognitions in case conceptualizations. However, the interplay between these metacognitions and dysfunctional coping strategies has not yet been sufficiently investigated in illness anxiety. Therefore, it seems sensible to attempt to better understand the mechanisms involved in the emergence and perpetuation of health anxiety. To increase our understanding, future studies should investigate clinical samples, manipulate metacognitions (e.g., 6) and examine their influence on SSB and AB. The potentially specific role of PMC for health anxiety and SSB has to be proven in studies comparing different clinical samples and studies which link specific metacognitions to different dysfunctional strategies (SSB, avoidance, hypervigilance). Finally, it would be of interest to examine, whether metacognitions explain incremental variance beyond and above other mechanisms relevant for pathological health anxiety (75), explain SSB and AB in day to day life and predict health anxiety longitudinally.“ (lines 453-468)

**Comment 14.** Even though no study assessed avoidance behavior, maybe the authors could explicit the theoretical and empirical links with health anxiety and metacognition?

**Reply:** In relation to your Comments 1-3, we have revised the introduction and detailed information on the most central constructs. We believe that this has also clarified the role of avoidance behavior and the links with health anxiety and metacognitions.

As described in the paper, there is a research gap regarding the association between the behavioral component of health anxiety and metacognitions. Especially avoidance behavior has barely been researched (Doherty-Torstrick et al., 2016) and is also neglected in the common assessment instruments for health anxiety (Weck, 2012). In existing studies, the extent of healthcare use has been mainly operationalized as a safety behavior (and avoidance of healthcare was not targeted often), or the focus has been placed on the related but more specific construct cancer worry (e.g., Jones et al., 2020). This makes it difficult to detail the theoretical and empirical links between avoidance behavior, health anxiety and metacognitions even more specifically than we have done so far. It is important for future research to investigate these connections to better understand health anxiety on a theoretical level and to derive specific treatment components, also considering which type of safety behavior the individual demonstrates (Furer & Walker, 2005; Jones et al., 2020). We hope our meta-analysis will help to shed light on this research gap and to encourage further studies in this field.

Jones SMW, Andersen MR, Litwin P. Avoidance and reassurance seeking in response to health anxiety are differentially related to use of healthcare. J Public Health (Berl.). 2022;30(2):475-480. doi: 10.1007/s10389-020-01299-8.

Furer P, Walker JR. Treatment of hypochondriasis with exposure. J Contemp Psychother. 2005;35(3):251-267. doi: 10.1007/s10879-005-4319-y.

**Minor comments and typos**

**Comment 15.** Formats of citations are inconsistent, some are author-date and others are numbers.

**Reply:** We aligned all citations meeting the Vancouver reference style.

**Comment 16.** Figures are separated from their captions.

**Reply:** We separated the captions from the Figures because this is a requirement of PLOS ONE (“Figure captions are inserted immediately after the first paragraph in which the figure is cited. Figure files are uploaded separately.“, see Submission Guidelines – Manuscript Organization on the PLOS ONE website <https://journals.plos.org/plosone/s/submission-guidelines#loc-figures-and-tables>).

**Comment 17.** The formatting of references is inconsistent. Please correct it.

**Reply:** Thank you, we reformatted the references accordingly.

**Comment 18.** In several places, punctuation is incorrect, complexifying the reading.

**Reply:** We proofread the whole manuscript to find errors in punctuation.

In conclusion, while this article tackles an interesting and important topic, the authors should clarify their research questions, better define their objects of study, and provide a more cohesive discussion of how these constructs relate. Greater detail about the relation of the present findings with the previous ones and existing theories of health anxiety would strengthen the contribution of this review to the field.

**Reviewer #2:**

Summary: In this systematic review and meta-analysis, the authors set out to assess associations between positive and negative metacognitions on the one hand, and health anxiety and related behaviors (safety seeking and avoidance) on the other. The study was preregistered. N = 23 studies were included in the final meta-analysis, with only few addressing safety-seeking and none assessing avoidance behavior. Results point towards medium-strong associations between metacognitions (both positive and negative) and health anxiety.

With this work, the authors provide a good and solid overview over the topic and are addressing a (surprising) lack in the literature. Their argumentation is consistently sound, and they reveal existing research gaps and point towards important future directions, both in the clinical and the research sphere.

A big strength of this work is its sound and transparent methodology, including the consistency with which the preregistration was followed. The paper is also very well written and was a true pleasure to read.

Therefore, I find the article basically ready for publication, although I would suggest fixing some super minor formulation/typo issues.

**Comment 1.** p. 3, l. 63: replace use of “Exemplarily”

**Reply:** We replaced “Exemplarily” with “As an example”. The sentence now reads as follows:

In the following citation, changed passages are highlighted in yellow: “As an example, a person might think that worrying helps with problem solving, motivates, prepares for eventual catastrophes, or prevents harm.” (lines 65-67)

**Comment 2.** p. 3, l. 74-76: replace (repeated) use of “next to”

**Reply:** We replaced “next to” in the paragraph with “in addition to” and “besides”. The paragraph now reads as follows:

In the following citation, changed passages are highlighted in yellow: “In addition to their relevance for GAD, metacognitions are supposed to specifically impact mental disorders that are characterized by any kind of intrusive thoughts (11). In line with this, besides GAD, especially large effect sizes of NMC were found for obsessive compulsive disorder and major depression when contrasting different psychopathologies meta-analytically (2). Moreover, metacognitive therapy has been proven to be an effective transdiagnostic therapy, superior to standard cognitive behavioral therapy (for a meta-analysis see 12).” (lines 78-84)

**Comment 3.** p. 16, l. 369-ff: the following sentence is perhaps a bit clunky and could be clarified: “These results underline the impact of NMC concerning the uncontrollability of thoughts, especially relevant for several different mental disorders (2) as well as for anxiety and depression across a variety of physical illnesses (57).” 🡪 what is "especially relevant", and what is meant by "across physical illnesses" (i.e., is this part meant to refer to instances where depression and anxiety co-occur with physical illness?)

**Reply:** Thank you for this important remark. We adapted the paragraph to make clear, that especially the facet ‘uncontrollability of thoughts’ in NMC plays a central role. And this applies to different mental disorders as well as to anxiety and depression co-occurring with multiple physical illnesses. The paragraph now reads as follows:

In the following citation, changed passages are highlighted in yellow: “These results underline the impact of NMC concerning the uncontrollability of thoughts. Consistent with Wells’ metacognitive model, NMC play a central role in several different mental disorders (2), as well as in anxiety and depression in the context of multiple physical illnesses (60).“ (lines 375-378)

**Comment 4.** p. 17, l. 410: missing comma before “others”?”

**Reply:** The comma before “others” was added.

**Comment 5.** p. 18, l. 424: rewrite “as adaptations of anyone inventory”?

**Reply:** This part was rewritten into the following:

In the following citation, changed passages are highlighted in yellow: “This is in line with studies finding that different health anxiety inventories (e.g., WI and (S)HAI, 70–73) often exhibit similarly high correlations, as do adaptations of any inventory (e.g., WI vs. WI-6, 56).“ (lines 427-430)

**Comment 6.** p. 18, l. 425-ff: something seems off in the following sentence (remove "as" or add something?): “However, as the calculation implies an equal weighting of all categories, which is perhaps not as meaningful as a qualitative description of the categories with increasingly high or low ROB.”

**Reply:** We changed the sentence a little bit to clarify our statement. The sentence now reads as follows:

In the following citation, changed passages are highlighted in yellow: “However, as the calculation implies an equal weighting of all categories, this result is perhaps not as meaningful as a qualitative description of the categories with increasingly high or low ROB.” (lines 431-433)

**Comment 7.** Supplementary/appendix: remove comment in S3 file and consider mentioning all supplements in the main text (as of now, no reference to S8 or S9, so their relevance is unclear)

**Reply:** Thank you. We removed the comment in the mentioned supplement file and added references to all supplement material in the main text. Please note that the supplementary material had to be renumbered accordingly.

**Comment 8.** Study selection: I was slightly confused by the information provided here as the number 23 refers to published studies, but then unpublished studies are mentioned, and it becomes also clear that not all data could be retrieved from the n =14 studies with missing data. So, are these selections that happen before the final number of 23 for analyses is established or do these numbers concern articles included in the 23 works selected for meta-analysis? When age averages are later reported it also becomes clear that not all studies are included here, so it might be best to provide an N of both studies and participants for calculated averages.

**Reply:** Thank you for bringing this to our attention. We adapted the paragraph in order to make it clear that the final sample of studies is 23. We changed the order of the information concerning sample sizes and added an additional sentence at the beginning. The adapted paragraph reads as follows:

In the following citation, changed passages are highlighted in yellow: “After the title-abstract screening, there were 51 studies sought for retrieval. Having access to 48 abstracts only, the authors of 3 studies were contacted to provide their full-texts. One study was still under review, another study was never published and the authors of the third study did not answer. There were 14 full-texts with missing or incomplete data reports. The authors of 5 studies sent us the relevant information. Concerning the other studies, the data were not accessible anymore (*n* = 4) or the researchers did not reply (*n* = 5). Finally, a total of 23 published studies were included in the meta-analysis. The PRISMA flow diagram (39) is shown in Fig 1. For an overview of the included studies see Table 1. Studies that were included in the meta-analysis but are not discussed in the paper can be found in the supplement (S4).“ (lines 249-257)

For the sample characteristics we added the number of participants for the age average (*n* = 8303) and age range (*n* = 7372). The N of the studies were already mentioned in this paragraph and for the other variables there were no deviating sample sizes or study numbers.

In the following citation, changed passages are highlighted in yellow: “In total, 9229 participants were included in 23 studies. The average age of the participants was 31.25 years (*n* = 8303, *SD* = 8.72*,* range: 16-80 years, *n* = 7372). Two studies did not include the average age, 7 studies did not report the age range. 5903 women (64%) and 3316 men (36%) participated. Twenty-one (91.30%) studies examined an analogue sample, one study (4.35%) recruited a clinical sample (self-reported hypochondriasis or illness anxiety disorder, 48), and one study (4.35%) assessed both (a clinically diagnosed sample, 11). The average level of health anxiety varied between the studies ranging from low to severely health anxious on the respective self-report scales. Often averaged health anxiety was mild. All mean values and standard deviations for the included studies can be found in the supplementary S5 Table.“ (lines 284-293)

Overall, congratulations to the authors for a great piece of work!
